# Assessing the restorative benefits of open rooftop green spaces characteristics using virtual reality and EEG analysis

**DOI:** 10.3389/fpsyg.2026.1807425

**Published:** 2026-05-20

**Authors:** Zhanglei Chen, Yanan Shen, Bing Zhu, Kar Kheng Gan, Tiejun Zhou, Yueyang Fang, Mingying Zeng

**Affiliations:** 1 School of Human Settlement, Mianyang Teachers’ College, Mianyang, Sichuan, China; 2 Sichuan Provincial University Engineering Research Center of Chuanxibei Rural Human Settlement (RHS) Construction, Mianyang, Sichuan, China; 3 Virtual Simulation Teaching Innovation Laboratory for Chuanxibei Human Settlements Center for Education Technology and Resource Development, Ministry of Education, Mianyang, Sichuan, China; 4 School of Economics and Management, Southwest Petroleum University, Chengdu, Sichuan, China; 5 TCL Studio, Adelaide, SA, Australia; 6 School of Architecture and Urban Planning, Chongqing University, Chongqing, China; 7 School of Civil Engineering and Architecture, Southwest University of Science and Technology, Mianyang, Sichuan, China

**Keywords:** electroencephalography, health design, open rooftop greening space, restorative environment, virtual reality

## Abstract

**Introduction:**

Open rooftop greening spaces (ORGS) offer a critical yet underexplored pathway to health restoration in high-density cities. While the general benefits of urban greenery are established, the specific environmental mechanisms and user responses within elevated, built-form-constrained spaces like rooftops remain poorly understood.

**Methods:**

This study systematically investigates how three key design variables—Green View Index, Sky View Factor, and functional type—independently and interactively influence multidimensional health outcomes. We employed a controlled virtual reality experiment integrated with electroencephalography and subjective scales to measure psychological, physiological, and social health responses among participants (*N* = 97).

**Results:**

Our results revealed a pattern of functional and environmental specificity in health benefits. Psychological relaxation and social well-being consistently peaked in environments combining high GVI with high SVF. In contrast, physiological activity willingness showed precise calibration: low-intensity activities (e.g., walking) were favored in high visual quality settings, while high-intensity exercise was uniquely promoted in fitness-oriented spaces with high GVI but lower SVF. Analyses delineated distinct roles for each variable. GVI emerged as the broadest predictor, particularly associated with social health and relaxation. SVF specifically facilitated psychological decompression. Functional type emerged as a key driver of activity intention but had minimal direct effect on social perception. Crucially, interaction models demonstrated that synergistic combinations are superior to maximizing single elements. The pairing of medium (not high) GVI with high SVF yielded the strongest psychological restoration, and high GVI was found to enhance the mental health benefits of dining/entertainment spaces

**Discussion:**

This study provides new quantitative evidence for the health-promoting potential of ORGS. The findings move beyond generic prescriptions, offering nuanced, evidence-based insights for salutogenic design. By leveraging specific visual-environmental synergies aligned with intended use, this work supports the design of rooftops that can contribute to urban mental resilience, social cohesion, and physical vitality.

## 1 Introduction

The modern conditions of metropolitan life, with all of its high density, permanent connectivity, and the saturation of sensory experience, provide a dual reality. Cities have unparalleled access to education, employment, and health care, which contributes to the development of society. Nevertheless, the same concentration tends to put residents at risk of chronical noise, air pollution, physical overcrowding, and, most importantly, lack of available natural spaces (Lachowycz and Jones, 2013). These factors, together with the inactive work habits, coupled with ongoing stress levels, are some of the factors that heighten the vulnerability of the population to non-communicable diseases (Corvalan et al., 2006). Often in dense urban cores, the constant stimulation is beyond the ability of the cognition to cope, resulting in overload. Chronic stress with a lack of rest is associated with poor concentration, emotional instability, and an increased incidence of anxiety and depression, so-called urban syndromes (Barton et al., 2015). The scale of such a problem is frightening: according to world estimates, there are about 450 million mentally ill people on the planet, and the traditional methods of response are based on clinical therapy and personal behavior transformation. Although they are required, these approaches tend to ignore the environmental and structural determinants of health. One of the most significant changes came with the Ottawa Charter of Health Promotion, published in 1986, which redefines health as something positive and reinforced by enabling environments and through the adoption of equitable policies, not as the lack of disease. This salutogenic re-invention of health as something positive, reinforced by enabling environments and equitable policies, has established urban design and green infrastructure as a key instrument of public health (Lovell et al., 2014). The ecological advantages of urban parks (Nordh et al., 2009), gardens (Reeve et al., 2017), and green corridors (Gobster, 1995) are unambiguous, and the psychological and social benefits of such intervention are proven and have been over time: stress relief, attention restoration, enhanced community bonds, and physical activity promotion (Hegetschweiler et al., 2017; Triguero-Mas et al., 2015; Matsuoka and Kaplan, 2008).

Rooftop greening is a timely and strategic intervention in this context. With space at the ground level limited in high population urban areas, construction of rooftops represents a large untapped resource (Johnston and Newton, 2004; Emilsson, 2008; Xu et al., 2012). Rooftop greening thus comes out not only as a form of compensatory green space, but as an initiative to gain back the ecological and social advantage of reclaiming the vertical aspect of the city, and it can be designed to convert roofs into usable green spaces and this makes the natural experience to become an integral part of the built environment since places of potential restorative experiences are at the center of everyday life where people work, live and interact. Incorporating nature into the city environment is an operational form of salutogenic infrastructure which can serve ecological sustainability, social integrity, and psychological resilience. Simultaneously, in two primary ways: visual experience and physical access (Williams et al., 2019; Oberndorfer et al., 2007; Loder, 2014; Shafique et al., 2018).

Existing Literature suggests that greening at high interfaces presents both different technical requirements, such as structural loading, drainage, insulation, and different concerns of user experience, participatory participation and aesthetic taste, all of which are context-dependent due to particular provision conditions (Ghosh et al., 2016; Hwang and Roscoe, 2015; General Services Administration, 2011; Blank et al., 2013). Subsequently, the restorative design paradigms based on traditional, earth-centered green space cannot be assumed to be transferable per se. It is against this backdrop, given the paucity of empirical knowledge on the subject, of material and perceptual features that define Open Rooftop Greening Space (ORGS) in China, that this investigation employs restorative environment theory to systematically interrogate its health effects, proceeding from the foundational premise that environmental perception is amenable to quantitative assessment.

Initial research on Open Rooftop Greening Space was largely descriptive, chronicling its implementation in cities like Singapore, Tokyo, and Sydney. These case studies established the multifunctional potential of rooftops, where vegetation was combined with dining terraces, exercise facilities, or cultural venues. A key early insight was that rooftops can go beyond ecological services like stormwater containment and urban heat islands and also offer psychological and social amenities (Rahman et al., 2015; Francis et al., 2017; Wolch et al., 2014; Tsantopoulos et al., 2018; Hunter et al., 2019). Through the provision of a third place that is unique out of home or work, these gardens were noted to reduce social isolation and create possibilities of informal leisure. As the example of Loder (2011) ethnographic study of North America showed, prairie-style green roofs as a third place lessened social segregation and offered opportunities to have informal leisure. The spaces were filled with wild grasses and flowering plants, which brought a feeling of fascination and curiosity to the users, which conforms to the Attention Restoration Theory (ART) concept of soft fascination. Even after making short visits, workers said they felt more refreshed and productive psychologically. Primitive experimental evidence was given by Lee et al. (2015), who found that attentional fatigue was significantly reduced by exposure to rooftop greenery than by the same exposure on sterile concrete roofs, so that ART was experimentally demonstrated to be empirically valid in a vertical context. On the same note, Mesimäki et al. (2019) affirmed that green roofs are restorative with the positive assessment associated with the visual quality, aesthetics, usability, and their capacity to arouse curiosity Although these works demonstrated that the popularity of green rooftops had a strong impact on perceived restorative value, Nagase and Koyama (2020) reported that turf roofs, which had the highest perceived popularity and potential health benefits, and biodiverse or solar-integrated roofs, which despite their greater ecological benefits, were considered to have lower restorative power because of their lower public familiarity.

Though these studies established that the popularity of green rooftops had a significant effect, they did not provide much information about the effect of specific, micro-scale features of the environment. To address this gap, subsequent studies, frequently based on controlled virtual environments, started to break down the role of individual vegetational features. Using photo simulations, virtual reality (VR) and controlled manipulations, researchers have been able to systematically separate particular environmental qualities that result in restoration. A representative study by Lee et al. (2014), comparing the preferences of 274 Australian office workers, made a classification of the rooftop vegetation based on structural properties, including the height of the plants, their life forms, and the color of the foliage. It was found that tall vegetation, flowering plants, and greenery that had the color predominantly green were always found to be the most restorative. On the other hand, vegetation with low growth and that with predominance of red color had the lowest scores on perceived restorativeness. These literatures highlight an important point, namely that the restorative impacts of greenery are moderated by the visual and structural characteristics of the plants in question, which in turn amplify perceptions of restoration. In the case of choice experiments, Trandafir (2023) established flowers, lawns, trees, and pathways to be the most preferred items on the rooftop. Collectively, these researchers found a stable preference of the population for rich, colorful vegetation designs compared to simple or thin designs, attributing the former to higher attractiveness and relaxation.

Regarding measurement, the restorative benefits of rooftop greening have been mainly measured in a subjective manner, such as structured interviews and questionnaires, which are effective in responding to instant post-exposure perceptions. Yuen and Hien (2005) were the first to apply these techniques in Singapore and provoke a traditional typology of the rooftops. They found that activities which promote the processes of restoration include children play, green exercise, psychological escape; Taib and Abdullah (2012) then narrowed them down into more specific categories such as the dining, fresh air, rest, landscape enjoyment, conversation and physical exercise; In the survey of Hong Kong rooftop landscape elements, Mak and Jim (2019) surveyed 477 residents, on the principal component and cluster analysis, perceptions of rooftop landscape elements were assessed. There were high preferences among the respondents towards leisure facilities (seating, kiosks, pathways), soft landscape elements (lawns, flowers, pergolas, hedges), and ornamental planting schemes with low vegetation and simple composition. On the same note, structured interviews of the stakeholders, such as designers, developers, property managers and residents, have confirmed that well-designed community rooftop spaces have a positive social atmosphere, perceptions of less crime, and a sense of belonging, although varying with the context of the built environment (Hadi et al., 2018).

Thus, while a substantial body of evidence confirms the positive public health implications of rooftop greening in cities, some scholars have begun to focus on its distinct restorative characteristics and mechanisms under the unique constraints of the built environment, differentiating it from traditional ground-level green spaces. A significant case is that of Mesimäki et al. (2017), who utilized deductive hypothesis tests plus imaginative descriptions offered by the participants to elicit the psychological imagery of the greenery on the roof. Their results indicated that open rooftop greening areas like the conventional ground-based urban green areas have the potential to foster social unity, multi-sensory experiences, and symbolic roles besides mellowing urban skylines. Notably, they also claimed that the targeted restorative processes may vary depending on the architectural attribute, roofspace function, scale and the main users expectation in which the rooftop is located in a relevant review, Williams et al. (2019) generalizes the different restorative processes of various urban green typologies. Some reviews posit that campus green spaces are mainly useful to improve student attention and academic performance, whereas residential green spaces help improve self-esteem, life satisfaction, and happiness due to the daily multisensory access. On the same note, street trees and urban forests have been observed to be effective in relieving depression, which is mostly facilitated by visual exposure. Within this functional range, Williams recognizes the main niche of rooftop greening to involve convenient mental and physical refreshing in momentary, intermittent breaks in the daily work pattern. This is in line with the idea of green micro-breaks as developed by Lee et al. (2018), which postulates that rooftop greenery can be used to influence the restorative effects that emerge more precisely using neurophysiological metrics. A research study carried out in Chengdu by Jiaxin (2023) has used the biometric measure of eye movement tracking, skin conductance, and heart rate variability. The discussion showed that there are great differences between three rooftop garden typologies namely plaza-park gardens, resting-stay gardens, and transit-passage gardens and their differences are mostly observed in such aspects of internal spatial attributes as scale, shape index, and richness of elements. The results showed that plaza-park gardens had better results in visual physiology (e.g., average pupil diameter, frequency of blink), emotional valence, and perceived dimensions of being away, fascination, and coherence. Conversely, resting-stay gardens had shown higher restorative effects in skin conductance response (SCR), heart rate variability (HRV), emotional arousal, and perceived “compatibility.” Transit-passage gardens demonstrated greatest positive effect in most of the metrics used like the average saccade frequency. Such evidence is a preliminary step to support the fact that various functional typologies of rooftop greening may trigger multiple restorative effect pathways across various dimensions of health.

Overall, the current studies have taken two directions: the shift towards the holistic environment to micro-elements and the shift towards subjective scales to neurophysiological indicators. While this progression has successfully identified a catalog of influential environmental attributes, the prevailing analytical paradigm has predominantly sought to isolate their independent, or net, effects. This trajectory has thus generated a catalog of potentially restorative attributes derived from ecological or aesthetic assessments of greenery (see, e.g., Fu, 2017; Nagase and Koyama, 2020; Jungels et al., 2013; Oberndorfer et al., 2007). However, as ORGS are increasingly conceptualized not merely as ecological patches but as a distinct form of public park in the vertical dimension, their restorative context diverges significantly from that of ground-level parks due to constrained area, microclimatic exposure, and often, more programmed use. This peculiar situation provokes important doubts regarding the applicability of design paradigms that are focused mainly on plant indicators. A predominant focus on isolated vegetation characteristics (Lee et al., 2015; Loder, 2014; White and Gatersleben, 2011).while foundational, may not fully capture the complex environmental gestalt of a rooftop, where factors such as spatial openness, functional zoning, and hardscape integration are equally constitutive of the user experience. Consequently, the prevailing analytical approach—seeking linear, net effects of singular elements—risks oversimplifying the interactive and contingent relationships that likely govern restorative outcomes in these hybrid spaces. This limitation is compounded by a primary reliance on subjective self-reporting, which, despite its value in capturing perceived restoration (Williams et al., 2019; Trandafir, 2023; Wolch et al., 2014; Tsantopoulos et al., 2018),lacks the convergent validation from physiological measures needed to robustly articulate the “how” behind health benefits in this specialized landscape typology.

To address these gaps, this study examines the restorative effects of open rooftop greening spaces through a VR-controlled experiment with neurophysiological monitoring (e.g., EEG) and subjective perception scales, measured in psychological, physiological, and social dimensions of health. This study therefore moves beyond examining factors in isolation, aiming to elucidate how they interact and combine to influence restoration. Building on this aim, this artical formulate the following testable hypotheses:

*H1*: The three restorative dimensions—psychological, physiological, and social—are expected to vary systematically across different functional types of ORGS. This variation would position functional programming as a primary organizing framework for restorative experience.

*H2*: The associations between key environmental characteristics and restorative outcomes are likely to be dimension-specific. That is, their associations are likely to differ meaningfully across psychological, physiological, and social health measures.

*H3*: The relationship between key environmental characteristics and restoration is contingent on functional typology. The restorative potential of specific environmental characteristics may therefore depend on the functional context of the space, pointing to the importance of synergistic design consideration.

The operationalization of these hypotheses is structured around three sequential research questions, designed to progress from descriptive mapping to the analysis of complex associations:

How are the psychological, physiological, and social restorative scores dispersed in different types of rooftop greening spaces?

How are the key environmental variables correlated with the different types of restorativeness?

How do these environmental variables interact with each other to affect the different restorativeness?

## 2 Method

This study was a mixed-method study that used a field-based survey of rooftop ecosystems coupled with an immersive virtual reality (VR) experiment on the basis of the restorative environment theory. Such a twofold method enabled acquiring both ecological validity, which captures the design and utilization of real rooftop spaces as designed and used in Chengdu, and experimental control, which allows manipulating the environmental variables systematically. The study aimed to ensure that the result was not just the perception self-report but also neurophysiological signs of restoration by combining both conceptual definition and on-site exploration in identifying representative samples (Section 2.1); Second, these samples were categorized and investigated with the help of field surveys, and, along with the knowledge gained in the literature concerning restorative environmental features, the key environmental factors that have the potential of restorative effects were singled out (Section 2.2); Lastly, 27 virtual scenarios were formed by systematically varying these factors between gradients to make an indication of different rooftop environments. The subjects had to undergo these situations in VR and record physiological responses with the help of EEG equipment (Section 2.3); the combined methodology had made possible a quantitative evaluation of restorative impacts of open rooftop greening space.

### 2.1 Study context and site selection

**Figure 1:**
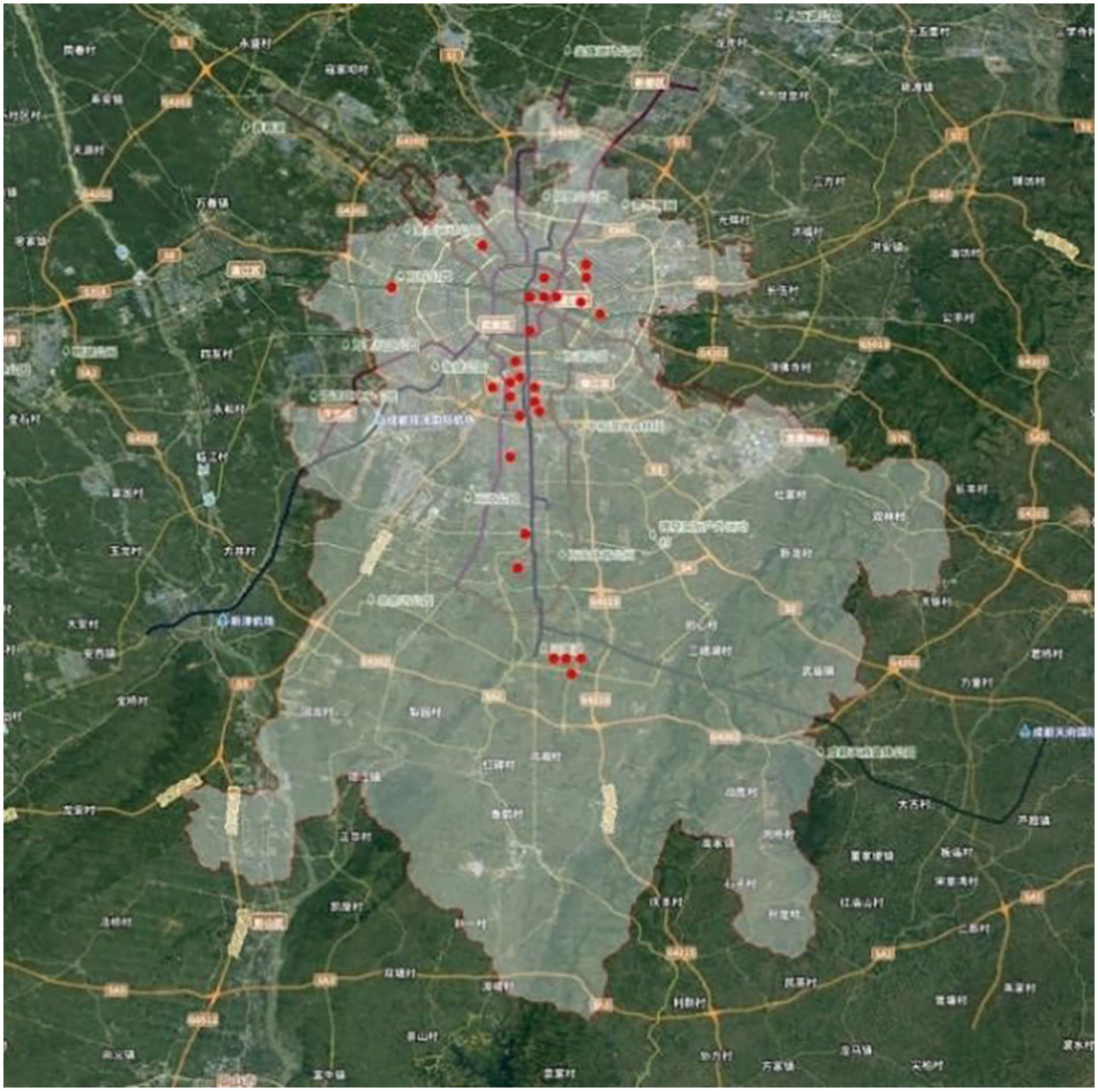
Site selection in the context of ChengDu.

Chengdu is a fast-growing Chinese megacity, and this paper empirically studies it. The city represents a salient setting in exploring rooftop greening as it is a densely-planned and supportive of progressive policies, as well as climatically-favorable setting. Chengdu has adopted a row of regulations and incentives to facilitate the process of three-dimensional greening since the early 2000s due to the desire to increase ecological resilience and the livability of urban areas. These initiatives have made it a national model in terms of large-scale, integrated development of rooftop landscapes. The humid subtropical climate of the city, with moderate temperatures and great relative humidity, enables the development of a wide variety of vegetation; the study area of this research was limited to an area of the Third Ring Road in Chengdu. This central urban area has a degree of development intensity of over 84.8, which is far above the international levels that indicate the extreme limitation on the development of new ground-level green space. In such a dense environment, rooftops become vital, under-used spaces, where natural vegetation is in a designed environment, where both ecological and social services are provided to the public to serve as sites of multi-purpose use, e.g., resting, socializing and informal recreation. ORGSs are separated by the mere technical roof areas (e.g., those that house mechanical equipment) or limited commercial terraces. They are conceptually placed in an intermediate position within the urban context: they are extensions of a building that help to improve the social face of a building and, at the same time, serve as interactive spaces that facilitate social interaction and help to shape the form of a city. The selection of the sample was conducted in a rigorous and multi-phase manner: site selection was done to make sure that the sample is representative. The preliminary desktop surveys and policy reviews were used to identify the possible locations in the study area. Subsequent field verification using three main selection criteria then resulted in the final sample of 28 different ORGS sites (see Figure 1 and Supplementary Table S1):

1. Unrestricted public access: sites must be freely available to the general public during operational hours, excluding venues with access limited to tenants, members, or paying patrons.
2. Multifunctional Design: Sites must support a variety of passive and social activities, thereby excluding single-purpose sports facilities or circulation-dominated areas.
3. Integrated natural-built character: sites must demonstrate a tangible integration of vegetation with human-scaled amenities like seating, paving, or shelters, excluding barren transit podiums or purely technical roofs.

The factors that determine the health benefits of environments are varied, which is based on the previous research findings using this curated sample to investigate the impacts that various environmental characteristics of rooftop spaces have on user experience and perceived restoration.

### 2.2 Extraction and establishment of experimental indicators

In order to draw the most applicable results to rooftop environments, the present research combined the results of both the literature on the restorative urban green spaces and on-site research on the construction of rooftop sites in Chengdu. Three environmental variables were thought of analyze them: the Green View Index (GVI), the Sky View Factor (SVF), and the functional type of Open rooftop greening space.

1. Green View Index (GVI): As opposed to planimetric measures (e.g., coverage ratio), GVI embodies the human experience of greenness, whereby it is more and more understood as a perceptually valid measure of the quality of urban greening. Since vegetation forms the most salient natural feature in rooftop settings, and previous studies indicated that GVI is strongly associated with restorative effects, this study adhered to accepted field procedures (Methorst et al., 2021).

To measure GVI, this study used the existing field practices. Within each site, sampling points were put in place within a grid of about 10 m x 10 m. Hemispherical photographs were made at every point towards the four cardinal directions (east, south, west, and north). Image analysis software was used to calculate the proportion of the area of green vegetation in each photograph, and the average of the four directions was taken as the GVI in that sampling place (Supplementary Table S2). Afterwards, the GVI data of the 28 rooftop samples were categorized into discrete levels according to known grades of the grading thresholds. Crucially, the specific numerical boundaries distinguishing ‘Low’ (10.54%), ‘Medium’ (19.84%), and ‘High’ (32.79%) levels were determined empirically. In a pre-experiment, a separate participant sample (*n* = 20) viewed VR scenes with a range of green vegetation proportions and rated their perceived ‘greenness.’ The GVI values that corresponded to statistically distinct clusters of these perceptual ratings were selected as the definitive experimental thresholds. Phytotypical planting structures were used as the models to test the hypothesis: The Low GVI grouping was simulated using a vertical structure of a shrub-grass with a majority of mixed evergreen and deciduous broadleaf communities. The medium GVI scenario utilized a tree-grass type of structure, which was dominated by evergreen broadleaf species. The more complicated layered structure of tree-shrub-grass was represented in the High GVI scenario, which was also based on the evergreen broadleaf communities. This quantitative index-visual-ecological archetype translation had the benefit of ensuring that the quantifiable experimental stimuli were accurate representations of the discernible gradients of greenery in real-life rooftop systems (Supplementary Table S3).

2. Sky View Factor (SVF): The Sky View Factor was used to measure visual openness and represented the ratio of visible sky to the total hemispherical vista. In dense urban areas, SVF also contributes greatly to the perception of enclosure and the spatial nature of outdoors environment. Due to the special exposure to the sky offered by rooftop spaces, unlike the ground level, SVF is a critical variable to evaluate the restorative capacity of rooftop spaces (Shooshtarian et al., 2018).

Computational analysis obtained the sky-visible fraction to calculate SVF (Supplementary Table S4). The sampled rooftops were also classified into three categories: Low (9.2%), Medium (17.4%), and High (27.5%). These percentage categories were calibrated based on perceptual data. A pre-experiment (n = 20) involving assessments of VR scenes with varying sky visibility established that these SVF ranges mapped reliably onto participant ratings of distinct ‘openness’ and ‘enclosure.’ Thus, the levels used in the main experiment were grounded in perceptible differences. These categories were scaled to experimental conditions by changing the presented height and distance to adjacent buildings to control the perceived level of openness of overhead space in the rooftop greening spaces (Supplementary Table S5).

3. Functional Typology: Rooftop greening spaces are elevated social spaces, in which the recreational functions seen on the common ground are inverted to the rooftop openness. Their practical organization, which is determined by the requirements of usability, social goals, and architectural incorporation, is a main factor of the activity pattern and the quality of the experience (Chen et al., 2022). The environment-behavior theory informed and field surveys in Chengdu confirmed the existence of three dominant functional models of rooftop greening spaces (Supplementary Table S6):

Sports and Fitness: Spaces suited to physical exercise (ex, running tracks, exercise stations).

Recreation and Sightseeing: Spaces suited to passive leisure, walking and landscape contemplation.

Catering and Entertainment: Spaces suited to dining facilities and structured leisure amenities.

Each of the three models was represented in the later experimental stimuli with typical furnishings and spatial arrangements to isolate the effects of functional programming on perceived restoration.

### 2.3 Experimental implementation and procedure

#### 2.3.1 Construction of virtual scene models

**Figure 2:**
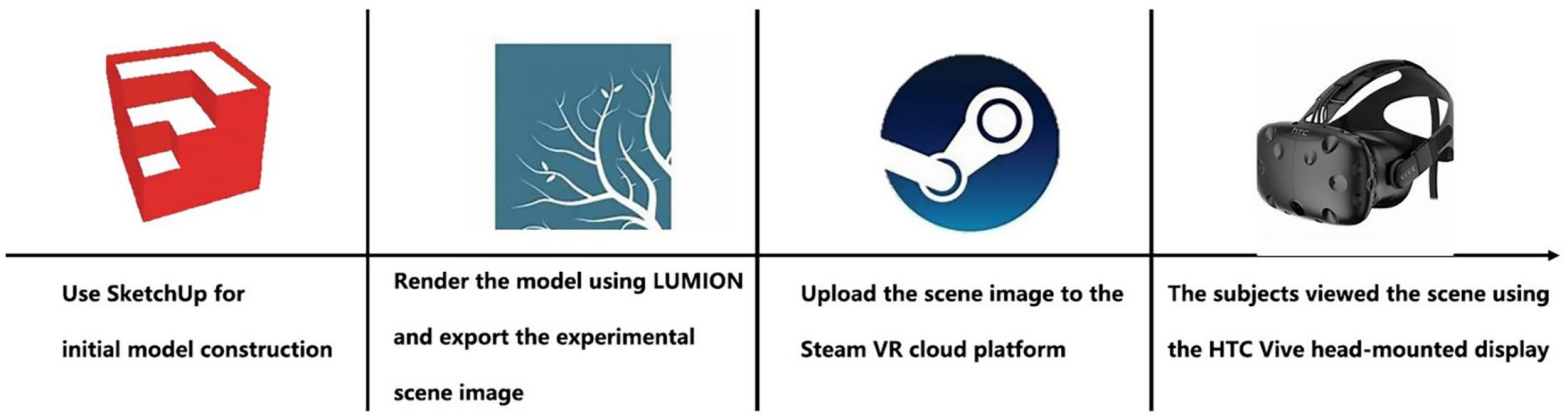
Virtual Reality equipment used in experiment.

The SketchUp 2020 three-dimensional base was put together based on a real and available podium-rooftop garden in Chengdu. This prototype site was chosen due to its typological prevalence; the field surveys showed that rooftops in the commercial districts, with a size of about 8,000 m 2 and enclosed structure, were the most prevalent. The regular geometry of the model also allowed the experimental manipulation of the model to be done accurately, and 360 panoramic images were also exported to Lumion 8.0 to add realism to the model. These panoramas were incorporated into SteamVR in order to provide a set of immersive visual experiences through the use of the HTC Vive headsets (see Figure 2).

The prototype was developed on the basis of a real open rooftop greening space in Chengdu, which is on the top of a six-story podium building that is enclosed all around by other surrounding buildings. The roof measured about 8,000 m ^2^ and was of a regular geometric design. Three significant environmental variables were systematically manipulated to develop the experimental stimuli based on classification indicators derived in the field (Supplementary Table S7). GVI and SVF were both modeled using the central 60° of the visual field which is representative of the normal human focal vision. A combination of all three variables at three levels provided 27 distinct virtual rooftop scenes (Table 1)

**Table 1:** Experimental model for virtual reality exposure.

Functional type	Green view index	Sky view factorC1	C2	C3
A1	B1	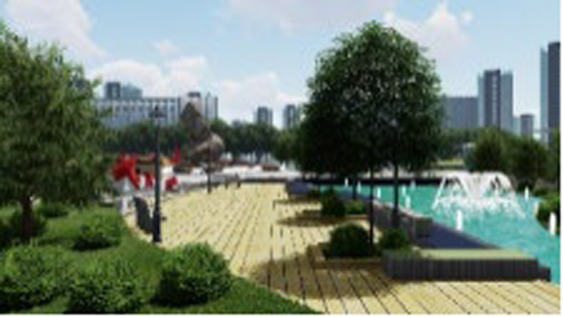	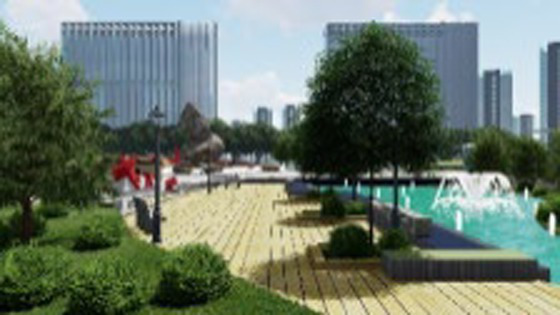	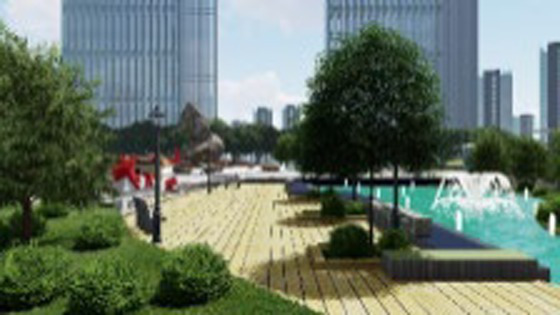
	B2	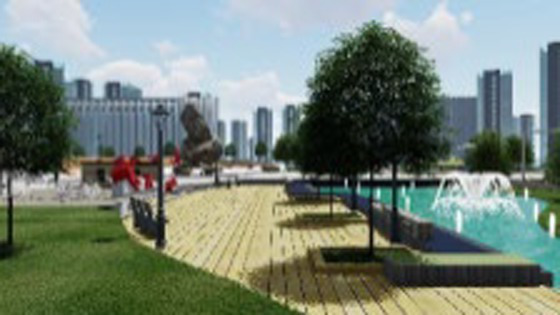	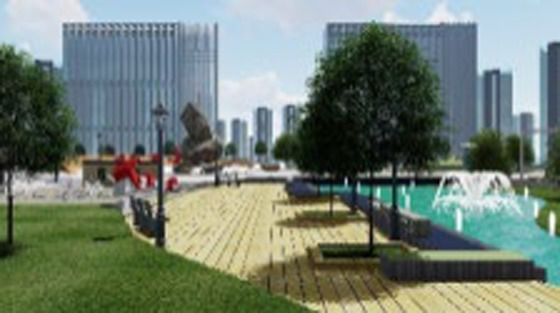	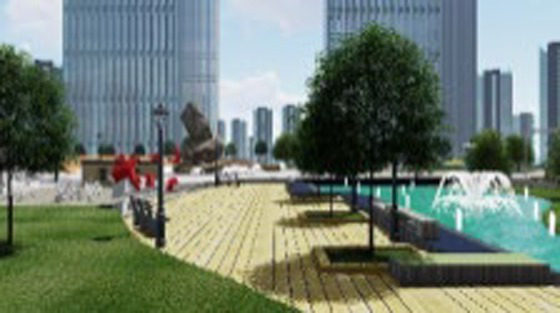
	B3	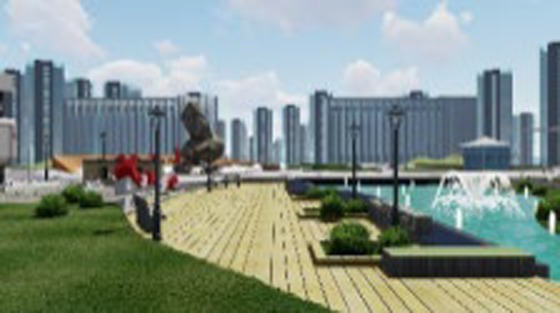	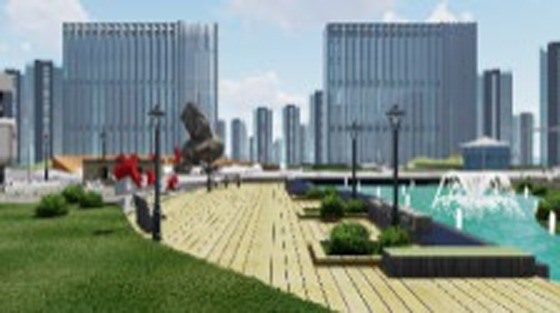	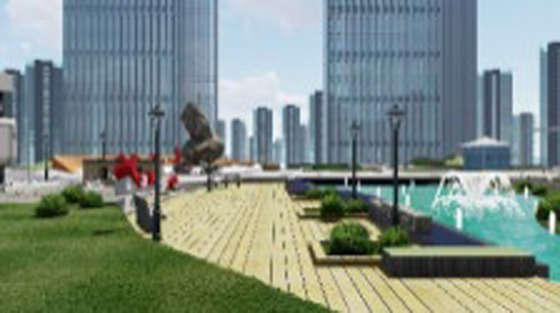
A2	B1	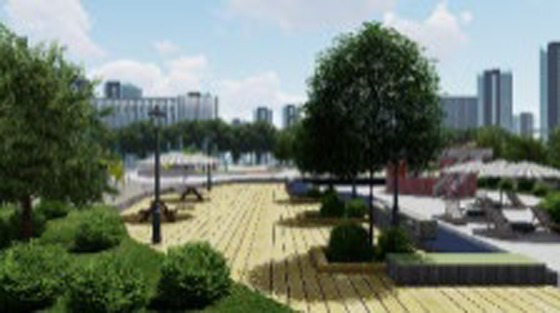	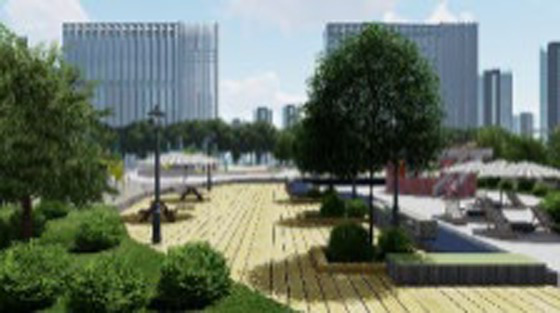	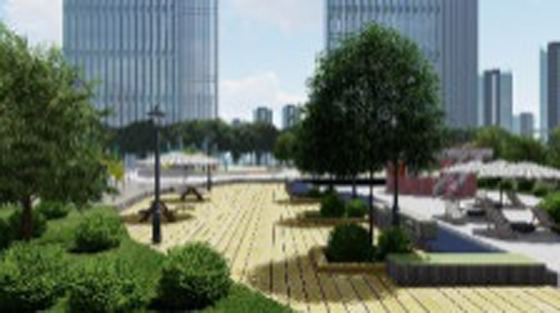
	B2	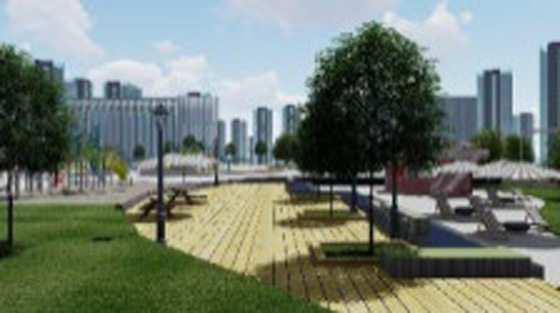	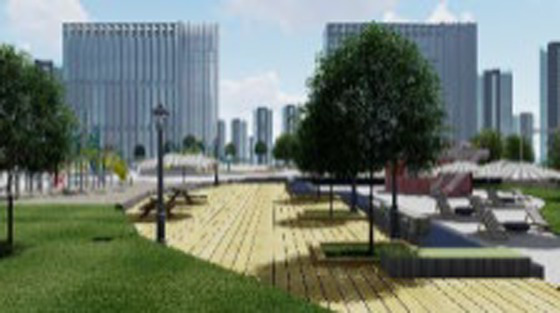	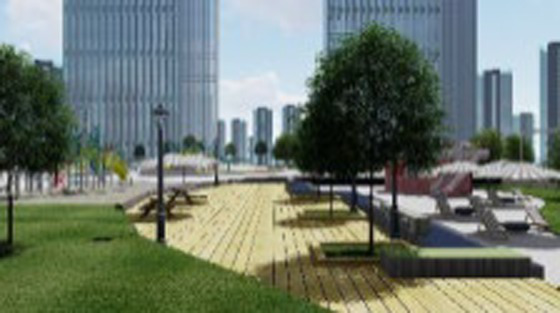
	B3	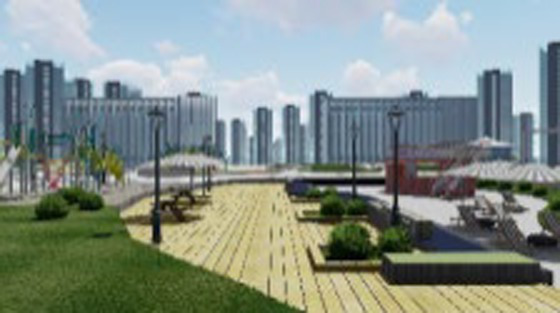	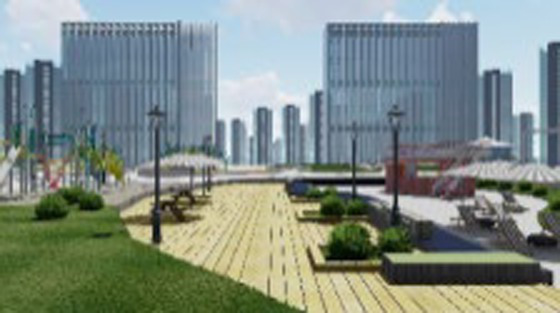	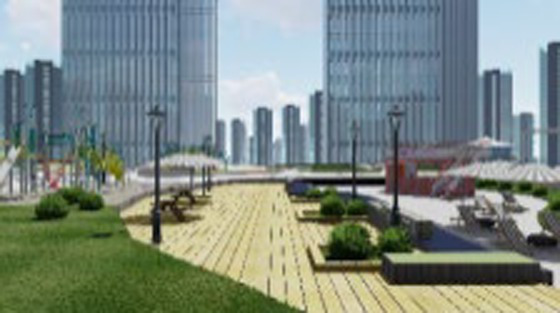
A3	B1	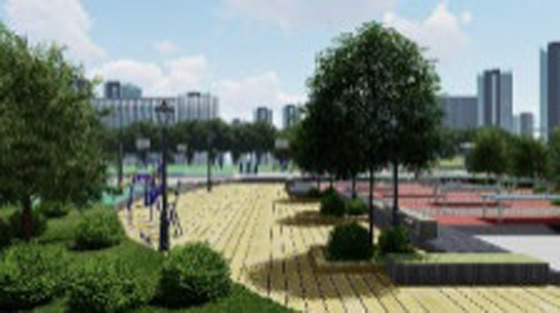	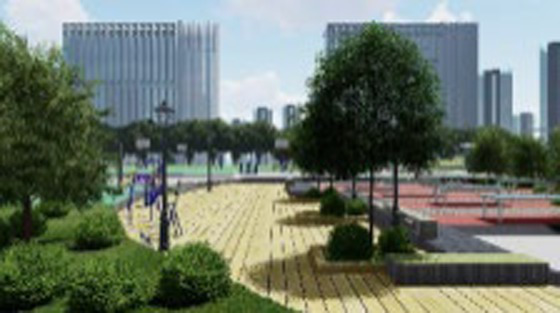	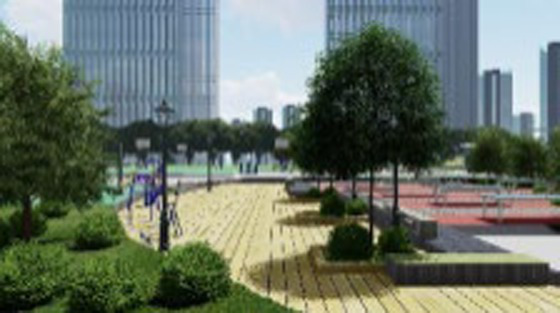
	B2	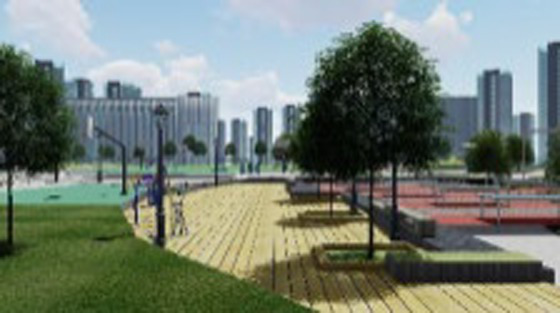	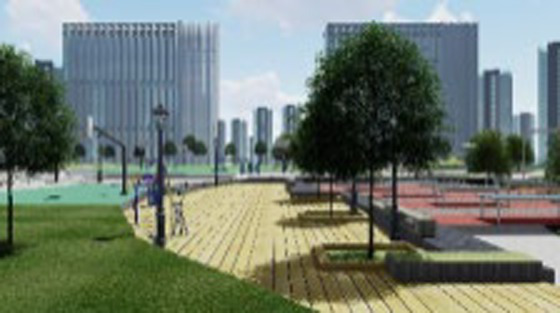	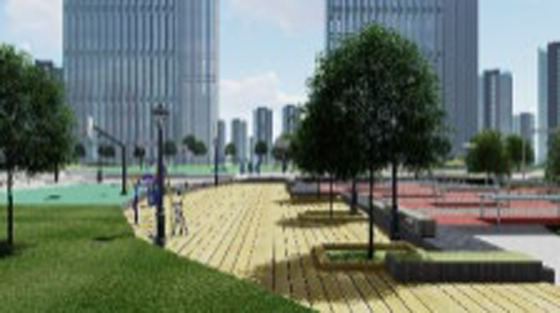
	B3	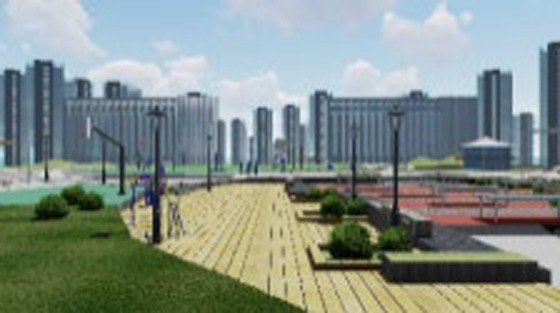	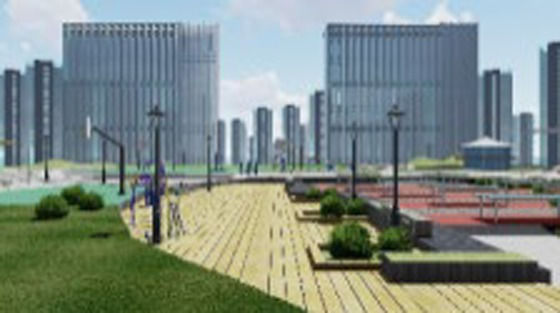	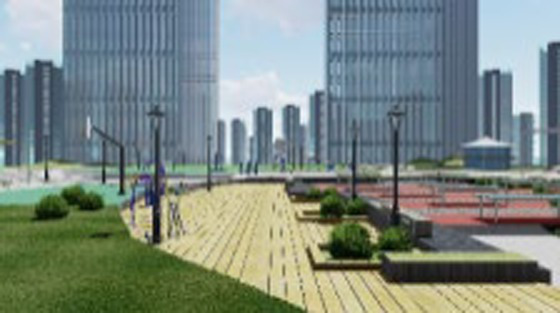

#### 2.3.2 Measurement of health indicators

To measure the responses of the participants towards the rooftop environments, an all-inclusive indicator system was developed, which included the following: psychological health benefits, physiological health benefits and social health benefits.

1. Psychological Health Benefits: Electroencephalography (EEG) provides a window into neural activity associated with psychological states relevant to restoration. Research in environmental neuroscience has established that EEG spectral patterns, such as alpha and beta band oscillations, are sensitive to exposure to natural versus urban environments and are correlated with self-reported measures of relaxation, attention, and stress recovery (Jiang et al., 2019; Zou et al., 2021; Mohamed et al., 2019; Goto et al., 2020). Building on this foundation, neural activity in this study was captured using a 14-channel EEG headset (Emotiv EPOC+). The raw signals were processed in real-time using the EmotivPRO software suite, which employs proprietary algorithms to translate complex spectral patterns into a set of six standardized affective and cognitive metrics: “Attention,” “Engagement,” “Excitement,” “Stress,” “Relaxation,” and “Interest.” These algorithms have been validated in prior studies against behavioral tasks and self-report measures, supporting their construct validity for assessing corresponding psychological dimensions (e.g., Al-Barrak et al., 2017; Aspinall et al., 2013). These derived indices served as complementary psychophysiological indicators of the psychological restoration elicited by the virtual environments (see Supplementary Table S8 for detailed definitions).
2. Physiological Health Benefits (Behavioral Intentions for Physical Activity) (hereafter measured as self-reported willingness to engage in specific activities): Research on environmental correlates of health often employs behavioral intention as a proximal indicator of a space’s potential to facilitate physical activity (e.g., Hegetschweiler et al., 2017; Hunter et al., 2019). To assess this potential within the ORGS contexts, participants reported their willingness to engage in six typical outdoor activities during a work break. These activities, identified via preliminary field surveys, included two dynamic activities (Walking, Exercise) and four static activities (Eating and Drinking, Viewing, Conversation, Reading). Willingness for each activity was rated on a 7-point Likert scale.
3. Social Health Benefits: The perception of social well-being was assessed on a composite scale adapted from de Vries et al. (2013), which measures five key dimensions: social connectedness, support, cohesion, quality, and welfare. In the present study, this composite scale demonstrated good internal consistency (Cronbach’s ɑ = 0.90). A total of 5 main perceptual dimensions were identified and transformed into a Likert scale questionnaire (see Supplementary Table S9).

#### 2.3.3 Experimental protocol

Participants were selected through Southwest University of Science and Technology through institutional announcements. The sample size used was a group of university students because, on two counts, they are an appropriate analog of the target population of stressed urban workers: first, they tend to be under a lot of academic stress; second, the basic patterns of visual perception and preference to the environment have considerable cross-demographic reliability and validity, which confirms that using students is valid and justified in the study of the population segment under investigation. They were asked not to drink alcohol, not to take caffeine, and not to go without sleep before the session. Individuals were excluded if they had underlying medical conditions, were taking relevant medications, self-reported a susceptibility to motion sickness, or reported experiencing persistent dizziness during preliminary VR exposure. Out of the 113 respondents, 101 of them consented to meet the criteria. Upon eliminating responses of four participants because of technical problems or missing responses, 97 valid datasets (55 male, 42 female) were stored, which is quite enough to perform statistical analysis of the results, but far enough to be considered as a typical set-up to study environmental perceptions. The experiment took place in a design studio which was converted to reproduce a generic high-pressure office setting. The process (outlined schematically in Table 1) was comprised of steps:

Pre-Test: The participants were informed and signed the informed consent, and completed a demographic and baseline stress questionnaire.

VR Adaptation: A 5-min period was offered to become acquainted with the VR headset. EEG Setup: The EEG headset was set up and checked. Participants were then verbally queried to confirm they felt comfortable and were not experiencing symptoms of cybersickness (e.g., dizziness or nausea). No participant reported significant discomfort requiring session discontinuation at this stage.

EEG Setup: The EEG headset was fitted and calibrated.

Baseline Recording: A 3-min quiet rest period established baseline neural activity.

Stress Induction: A 3-min cognitive stressor task (involving verbal and arithmetic exercises) was administered to elevate arousal. Following this task, neurophysiological (EEG) data were recorded for 1 min to capture the elevated arousal state. Participants were then given a brief, standardized rest period until their self-reported calmness and real-time physiological readouts (monitored via the equipment) indicated a return to a stable, pre-stimulus resting baseline, ensuring that subsequent VR exposure started from a consistent physiological state.

Recovery/Exposure: The participants were shown the 27 virtual rooftop scenes in randomized order. The scenes lasted 40 s each- a time that was proven to induce micro-restorative effects. Right after every scene, participants evaluated the willingness of activities and social perceptions during a 40-s response time. To minimize carryover effects between trials and allow for momentary recovery, each 40-s exposure epoch was structured as follows: the first 20 s consisted of active viewing, after which a snapshot of the neurophysiological data was recorded; the remaining 20 s served as a short, eyes-closed rest interval before the next scene was presented. Furthermore, the total session was divided into two blocks with a mandatory 5-min seated rest period in between to counteract fatigue. The total length of the session was about 50 min per subject.

#### 2.3.4 Data preprocessing and preliminary analysis

**Figure 3:**
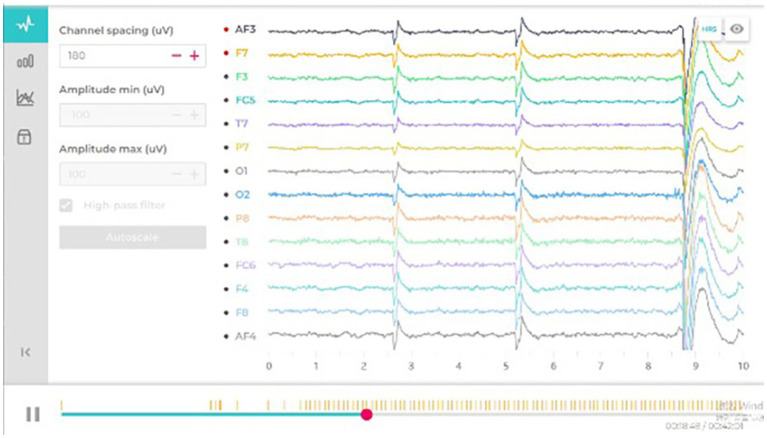
Changes in basic EEG data.

**Figure 4:**
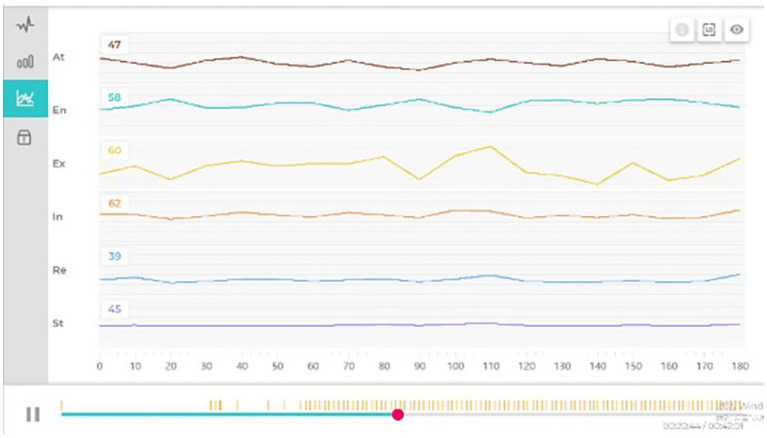
Changes in final EEG data.

EEG signals (14 channels) in the raw form (sampling rate of around 128 Hz) were analyzed using the Emotiv software to extract the six psychological variables. These values were averaged across 10-s epochs. Noise or movement artefacts were determined and omitted (see Figures 3, 4).

Mean values of each measure were estimated at three main stages: Baseline, Stress Induction and VR Exposure. To directly quantify the restorative shift, our primary analysis focused on change scores computed as the difference between the VR Exposure phase and the Stress Induction phase (VR-Stress) for each index. This approach provides a measure of psychophysiological recovery from the induced stressor. The comparison of the Stress and VR phases was based on the analysis in order to measure change. We interpreted increased “Attention,”” Engagement,” “Excitement,” and “Stress” scores as markers of elevated cognitive load and arousal associated with the stressor task. Conversely, increases in “Relaxation” and “Interest” were interpreted as indicators of reduced arousal and positive emotional shift, signifying restorative progress.

Given the repeated-measures design, the resulting epoch-level data for each outcome variable were inherently non-independent within each participant. To meet the assumption of independent observations required for subsequent group-level inferential statistics (e.g., ANOVA, regression), the data were aggregated prior to these analyses. For each participant and experimental phase (Baseline, Stress Induction, VR Exposure), all valid epoch-level values for a given measure were averaged to produce a single, representative data point per phase. Consequently, all reported statistical tests were conducted on this aggregated dataset, where the unit of analysis was the participant-phase mean (*N* = 97 participants × 3 phases = 291 independent observations). This aggregation process mitigates the risk of inflated Type I error by eliminating within-subject correlations from the dataset used for hypothesis testing.

**Figure 5:**
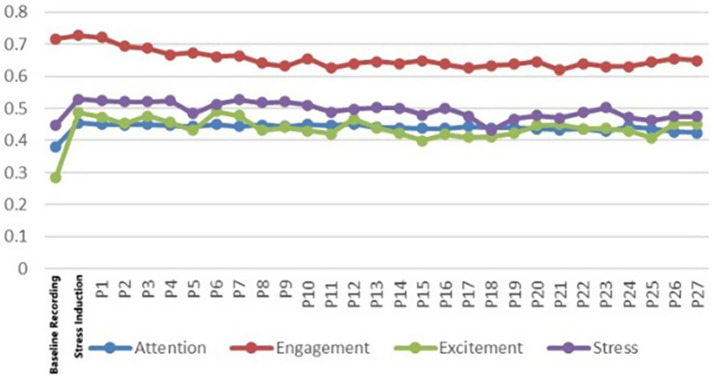
Changes in the final negative data of the pre-experiment.

**Figure 6:**
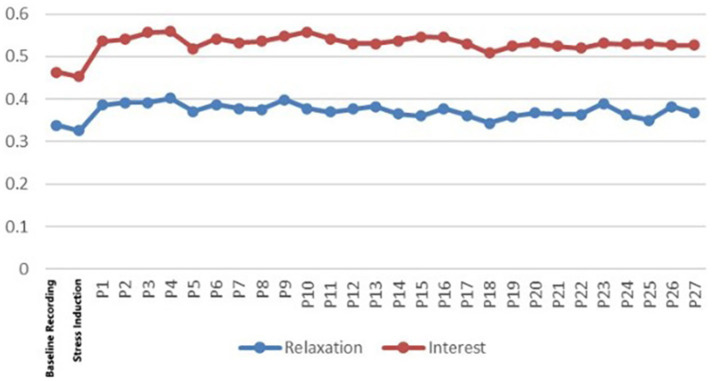
Changes in the final positive data of the pre-experiment.

The analysis of the EEG patterns in the 27 sampled open rooftop greening spaces in Chengdu confirmed the anticipated pattern: measures of cognitive load reached their highest point during the Stress phase and declined during VR exposure and Relaxation and Interest scores during the Stress phase and bounced back during the VR exposure (see Figures 5, 6). This pattern provides initial evidence that the virtual rooftop stimuli elicited psychophysiological shifts consistent with restoration.

Prior to inferential analysis, the distributional properties and key parametric assumptions for all aggregated outcome variables were examined. The psychological measures comprised six continuous indices derived from EEG spectral analysis. The activity willingness and social health measures were based on multi-item Likert-type scales; their mean scores were treated as continuous variables for analysis, a common and justified approach when scale points are multiple and distributions approximate interval-level properties. For all variables, univariate normality was assessed using skewness and kurtosis statistics. The absolute values of skewness were all below 0.86, and those of kurtosis below 0.48, indicating no substantial deviation from normality and supporting the use of parametric methods (West et al., 1995). Potential univariate outliers were identified as data points exceeding ±3.29 standard deviations from the variable mean (Tabachnick and Fidell, 2013). Fewer than 0.93 of all data points met this criterion. As these outliers were not systematic (i.e., not concentrated in a specific experimental condition) and their exclusion did not alter the significance or direction of the primary findings, they were retained to maximize statistical power. Furthermore, the assumptions of homoscedasticity (verified via Levene’s test, all *p* > 0.05), linearity (confirmed via visual inspection of scatterplots), and absence of multicollinearity (all variance inflation factors < 1.93) were satisfied, thus validating the subsequent use of ANOVA and linear regression models

#### 2.3.5 Statistical analysis plan

The analytical process was developed in a sequential manner to answer the three main research questions. First, to address the initial question regarding the distribution of health outcomes, descriptive statistics—primarily mean values across experimental groups—were calculated. This step presented a preliminary summary of the central tendency of psychological, physiological, and social measures of health in the presence of various functional type, GVI, and SVF conditions and the dimensions of health that appeared to be most pronounced in particular environmental conditions.

Proceeding to the second research question, which concerns the relationship between environmental predictors and health outcomes, a two-stage procedure was implemented. Pearson correlation (for continuous GVI/SVF) and one-way ANOVA (for categorical functional type) were first applied to identify variables with statistically significant bivariate associations (*p* < 0.05). Given the exploratory aim of identifying which fine-grained health sub-indicators were most sensitively linked to each environmental factor, this screening step served to reduce noise and prevent overfitting. By focusing subsequent regression on the most robust linear signals, we enhanced the parsimony and clarity of reporting for H2, compared to fitting a full set of 18 complex models containing potentially non-significant terms. These significant predictors were then entered into multiple linear regression models. Within these models, the standardized regression coefficients (*β*) were interpreted as indicators of the magnitude and direction of each environmental variable’s unique, net influence on a given health outcome, after accounting for other variables in the model.

Lastly, to test the presence of synergistic effects as in the third research question, the regression framework was extended. For this explicitly theory-driven analysis, the vision shifted the unit of analysis to composite scores representing the overarching health dimensions. This ensured that the test of interactions was comprehensive and not constrained by the prior variable selection focused on sub-indicators. Two-way interaction terms (e.g., GVI × Functional Type) were added to the models. The statistical significance of an interaction term indicates whether the effect of one predictor meaningfully changes across levels of another. Furthermore, the coefficient for a significant interaction term quantifies the incremental effect of that specific environmental combination, relative to the additive effect of the individual variables alone. This enables the determination of certain design configurations that produce enhanced restorative effects.

## 3 Results

### 3.1 Descriptive analysis of psychological, physiological, and social health characteristics in open rooftop greening spaces

This section provides a foundational descriptive analysis (i.e., comparison of mean values) of psychological, physiological, and social health indicators across the 27 experimental scenarios, which were constructed by combining the three fundamental environmental indicators: Green View Index (GVI), Sky View Factor (SVF), and functional type. The specific thresholds defining ‘Low’, ‘Medium’, and ‘High’ levels for GVI and SVF were established through site analysis (see Methods 2.2 and Supplementary Tables S2–S6). The following analysis describes notable patterns in the mean scores across these conditions. All references to ‘higher’ or ‘lower’ scores are based on observed mean differences for descriptive purposes and are not outcomes of inferential statistical tests.

#### 3.1.1 Psychological health effects

**Figure 7:**
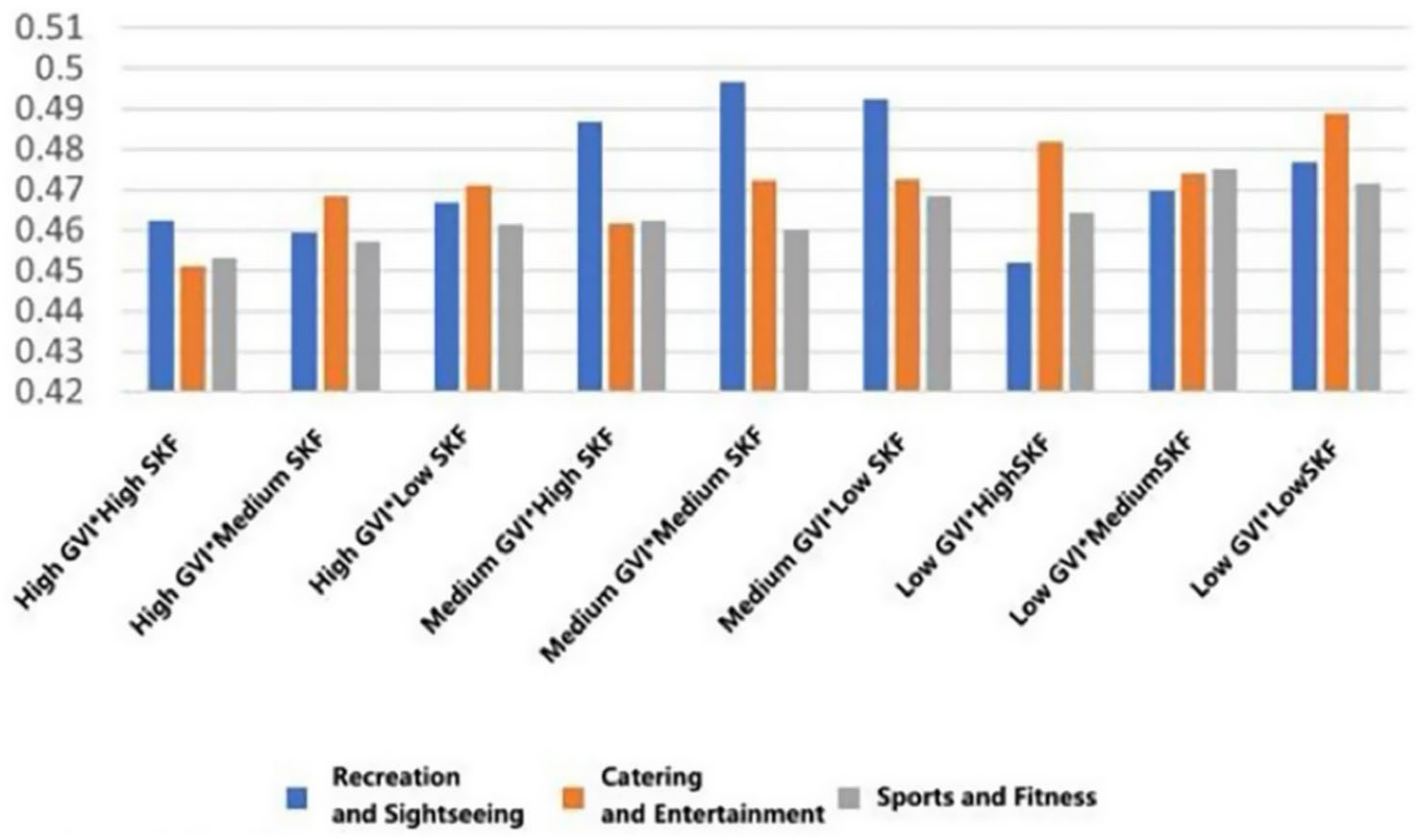
Mean changes in attention under different environmental characteristics.

As shown in Figure 7, the mean attention values, based on EEG recordings, would be varied across conditions. In recreational rooftops, low GVI (10.54%) and high SVF (27.47%) were correlated with the lowest observed mean score for attention (M = 0.451, SD = 0.074), which reflects a state that cannot be characterized as demanding directed mental activity.

**Figure 8:**
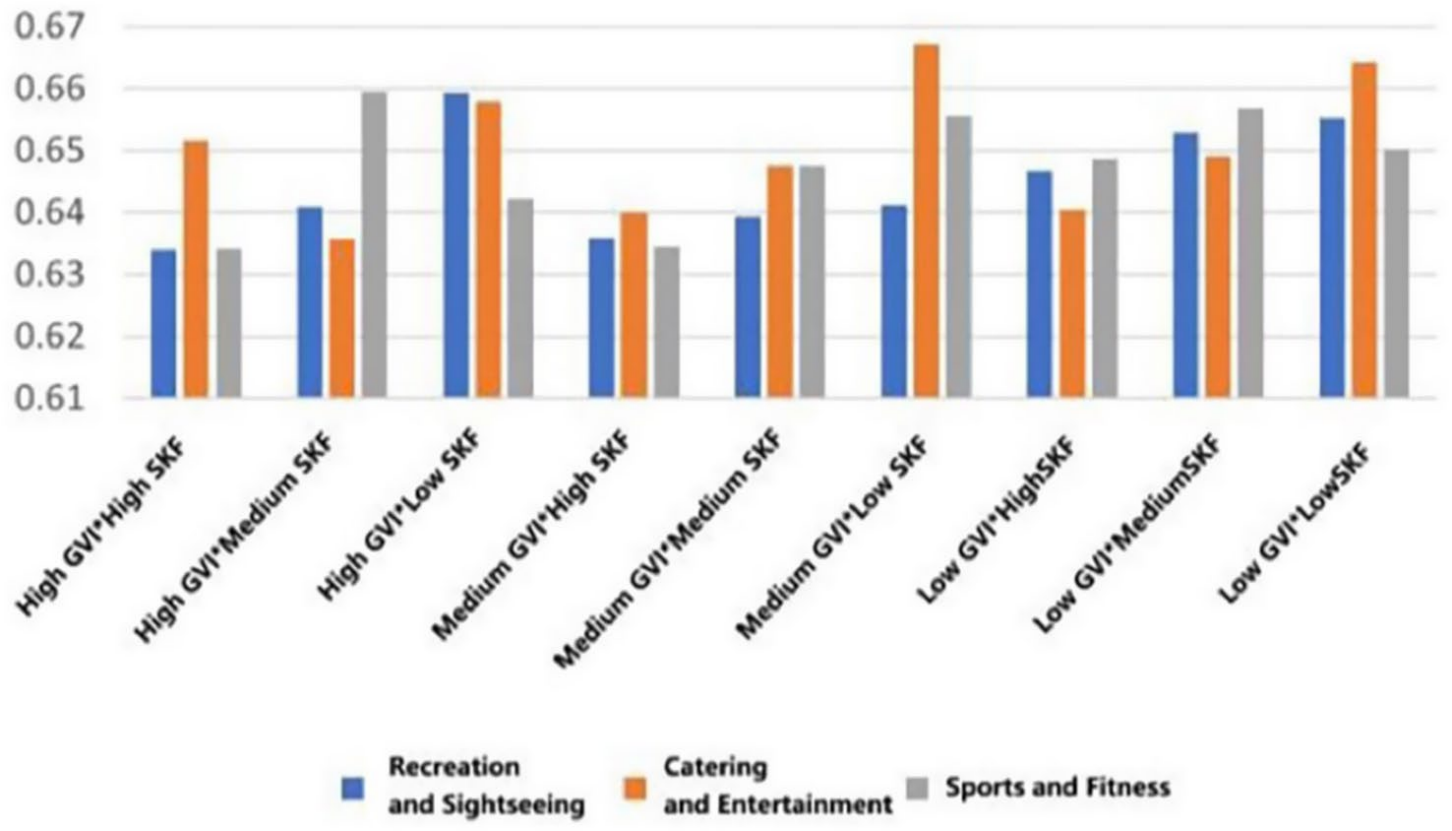
Mean changes in engagement under different environmental characteristics.

The same pattern was observed with regard to the levels of engagement (Figure 8). Rooftops with high GVI (32.79%) and high SVF (27.47%) had the lowest mean engagement score in recreational rooftops (*M* = 0.633, SD = 0.087). The same could be said about entertainment-type rooftops, as high GVI and medium SVF (17.43%) elicited equally low engagement (*M* = 0.634, SD = 0.116). though the difference was not as high in the case of fitness-type rooftops, the same trend was observed, with high GVI and high SVF being associated with low engagement (*M* = 0.633, SD = 0.087).

**Figure 9:**
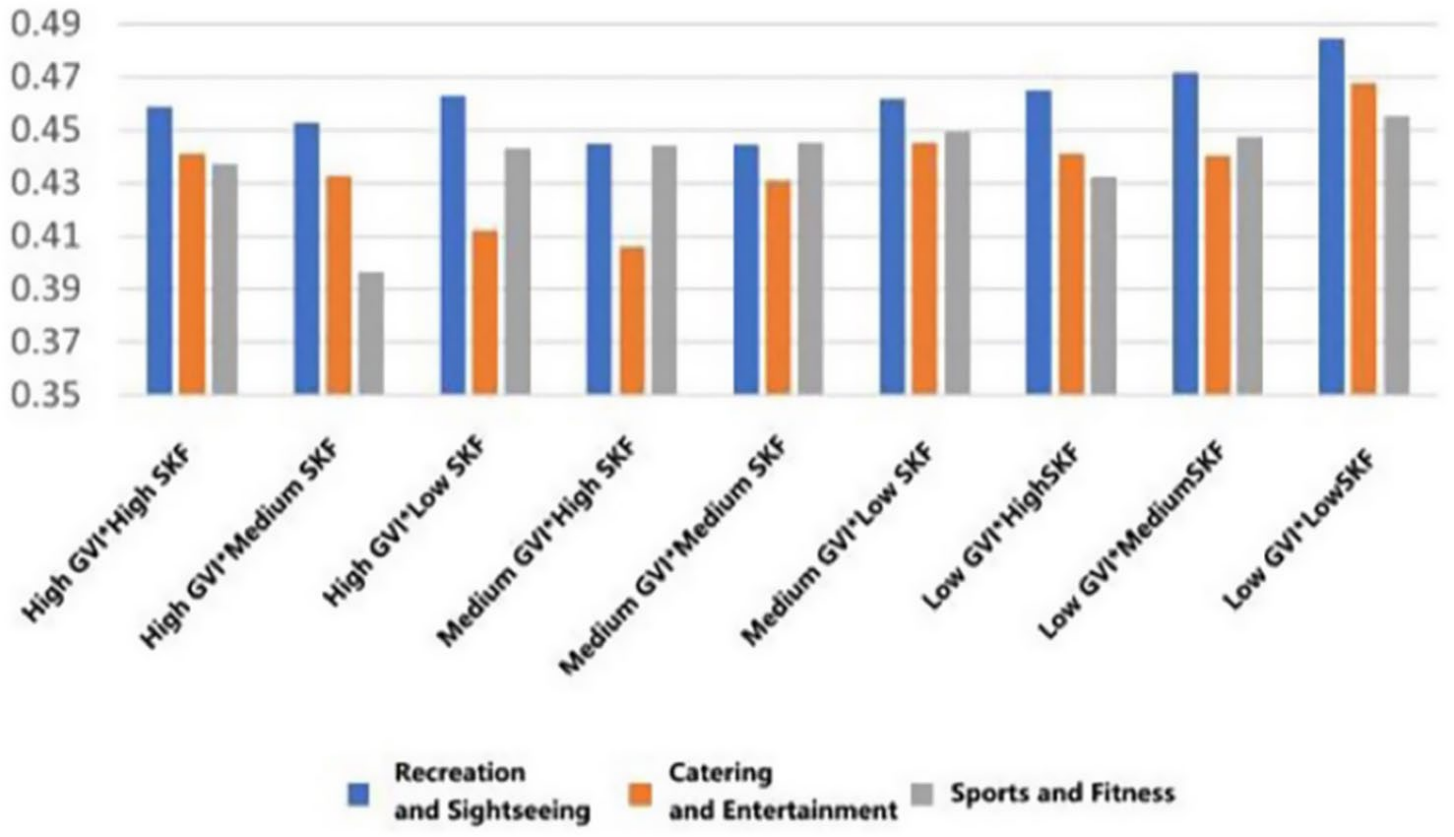
Mean changes in excitement under different environmental characteristics.

**Figure 10:**
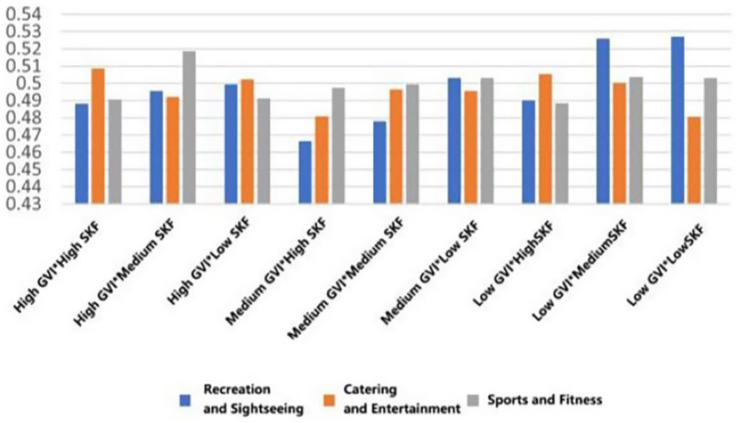
Mean changes in stress under different environmental characteristics.

Mean excitement levels (Figure 9) were lowest in the case of entertainment-type rooftops with medium GVI (19.84%) with high SVF (M = 0.407, SD = 0.192) Concurrently, perceived stress (Figure 10) was most effectively alleviated (i.e., showed the lowest mean score) in recreational rooftops configured with medium GVI (19.84%) and high SVF (*M* = 0.468, SD = 0.176).

**Figure 11:**
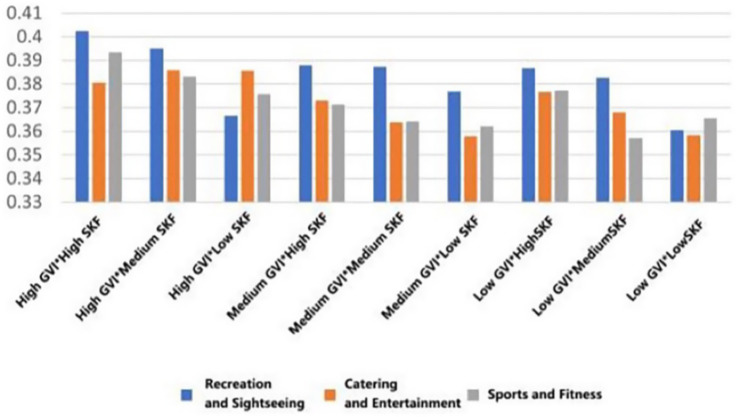
Mean changes in relaxation under different environmental characteristics.

**Figure 12:**
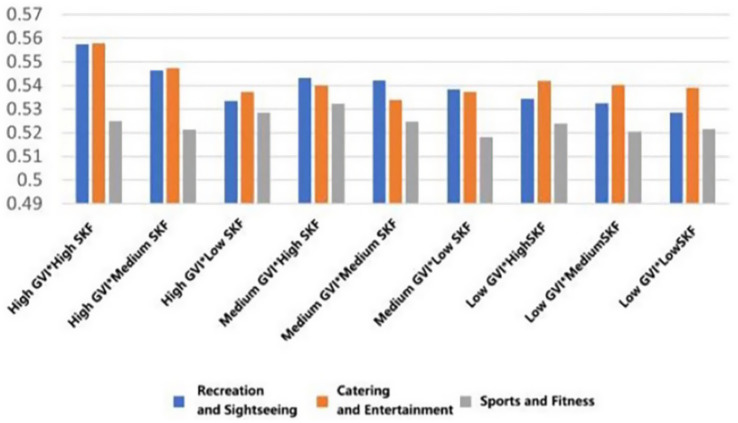
Mean changes in interest under different environmental characteristics.

Mean relaxation measures (Figure 11) showed a positive and consistent response with greater greenery and openness of the environment. Recreational rooftops that had high GVI and high SVF showed the highest scores (*M* = 0.402, SD = 0.166). This was closely followed by entertainment rooftops with high GVI and medium SVF (*M* = 0.384, SD = 0.151), and fitness rooftops with high GVI and high SVF (*M* = 0.393, SD = 0.166). The same pattern was reflected in interest levels (Figure 12), peaking with high GVI and high SVF conditions in both recreational and entertainment settings (*M* = 0.599, SD = 0.119).

#### 3.1.2 Behavioral intentions for physical activity

**Figure 13:**
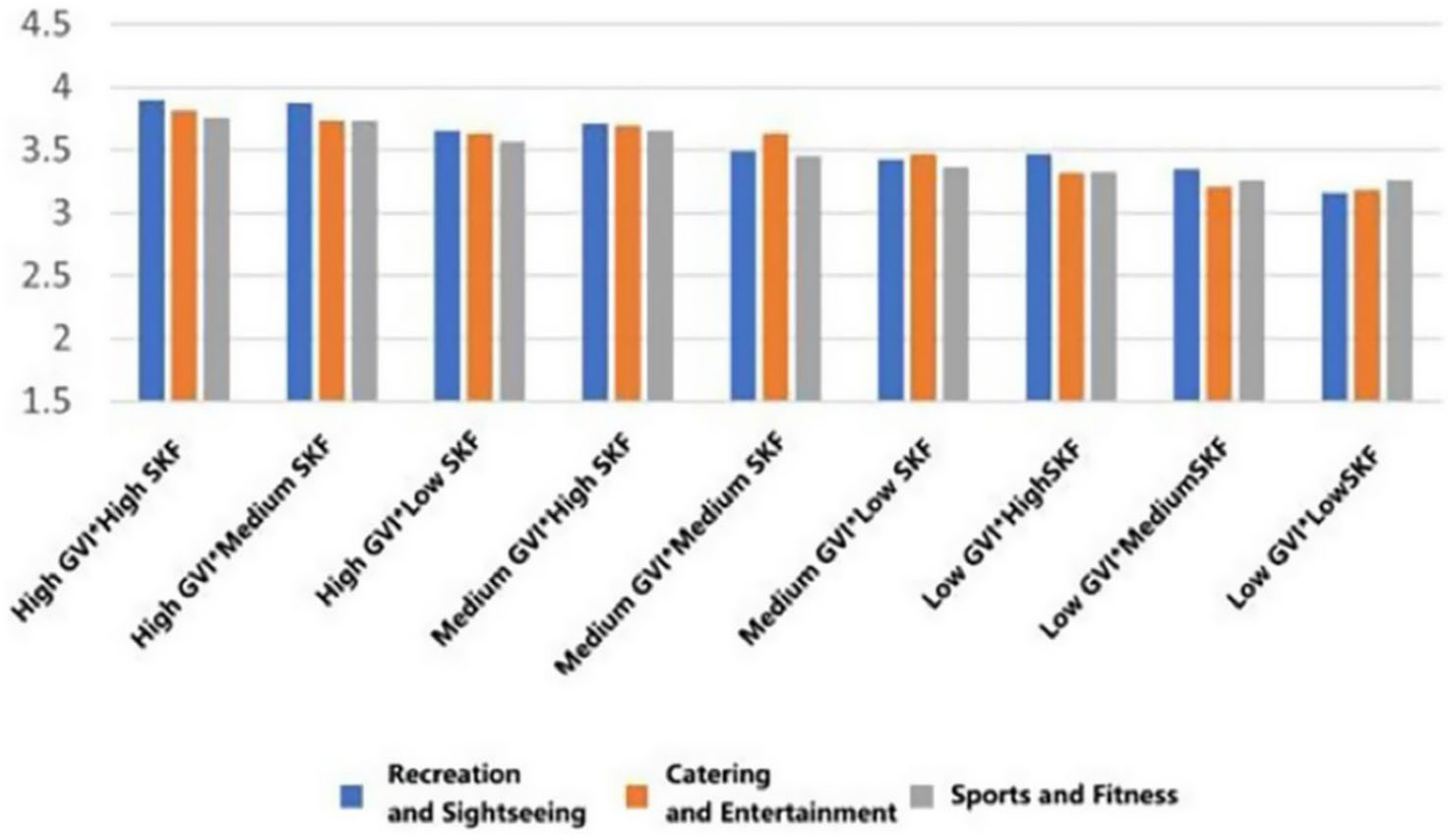
Mean changes in walking under different environmental characteristics.

With the highest mean preference to walk in the conditions of high GVI and high SVF in all types of functional settings (*M* = 3.891, SD = 0.699; *M* = 3.730, SD = 0.713, *M* = 3.652, SD = 0.799). On the other hand, the worst scores on willingness were achieved in low GVI (10.54%) and low SVF (9.19%) conditions (*M* = 3.183, SD = 1.029, *M* = 3.260, SD = 0.934, *M* = 3.431, SD = 1.031). This linear trend indicates that the co-presence of abundant greenery and an open sky most effectively promotes restorative ambulation (Figure 13).

**Figure 14:**
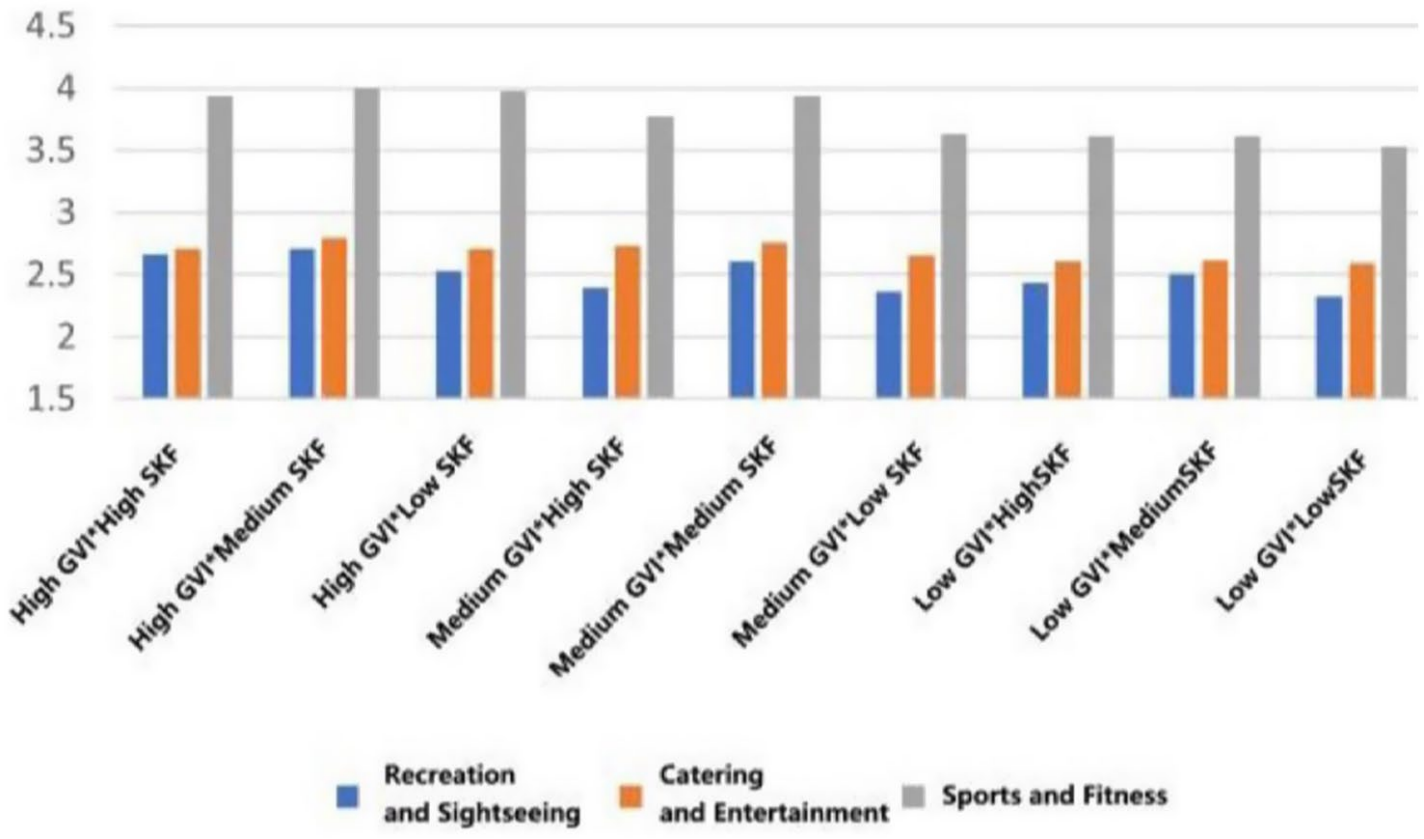
Mean changes in exercise under different environmental characteristics.

For dedicated exercise, peak mean willingness was exclusive to fitness-oriented rooftops, occurring with high GVI paired with either medium or low SVF (*M* = 4.001, SD = 0.825/0.834). The most unfavorable mean background of exercise was once again the low GVI and low SVF combination (*M* = 3.501, SD = 0.919). This trend suggests that in the case of focused physical training, visual greenery is important, but to some extent, spatial enclosure (lower SVF) can also help to concentrate (Figure 14).

**Figure 15:**
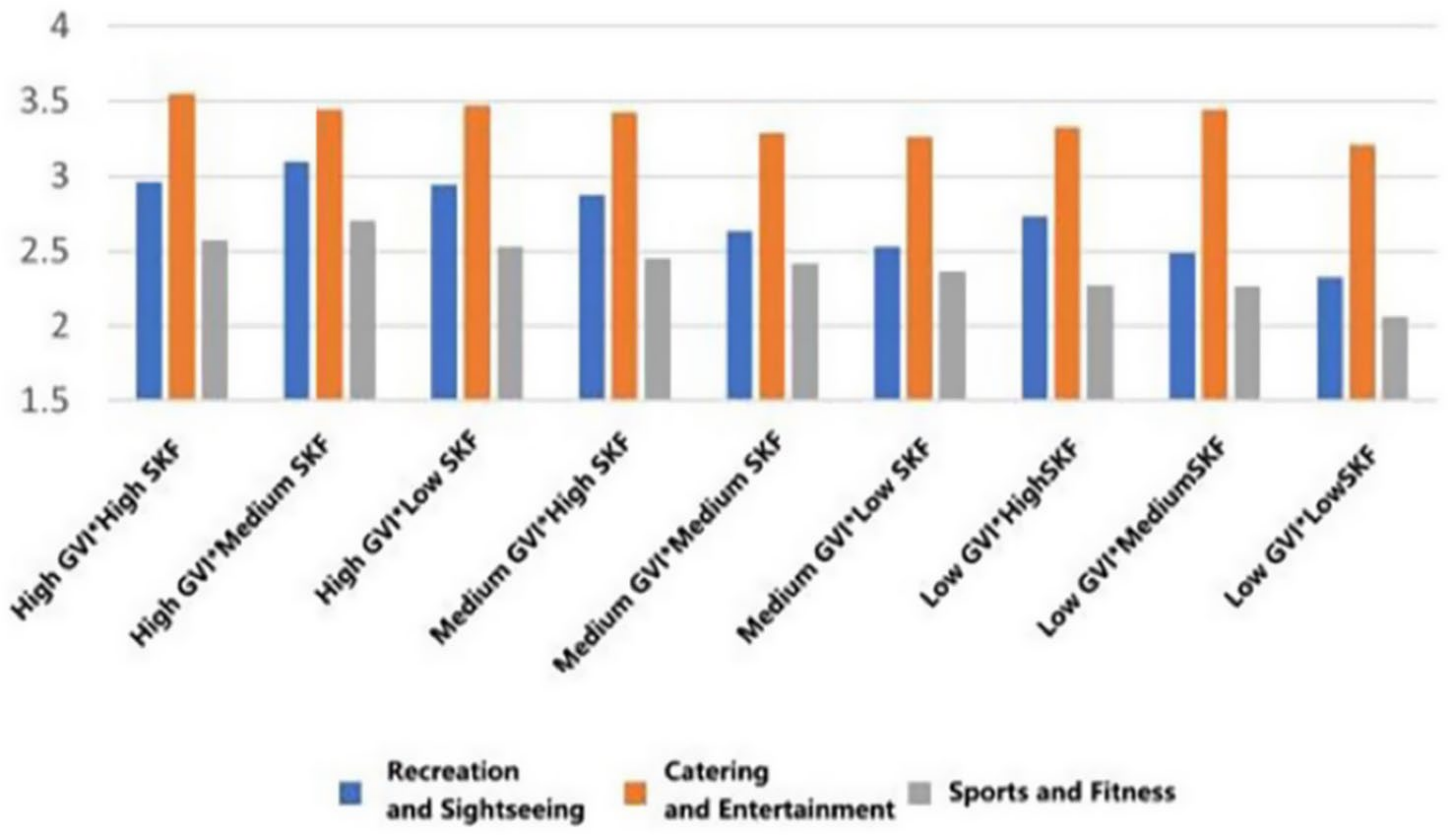
Mean changes in eating and drinking under different environmental characteristics.

A different trend was found in general leisure activities. The mean willingness stayed at a similar level in the rooftops of the entertainment type (M = 3.494, SD = 0.914), and was not much affected by the variations in GVI and SVF. This indicates the high intrinsic attractiveness of the social-recreational program *per se* (Figure 15).

**Figure 16:**
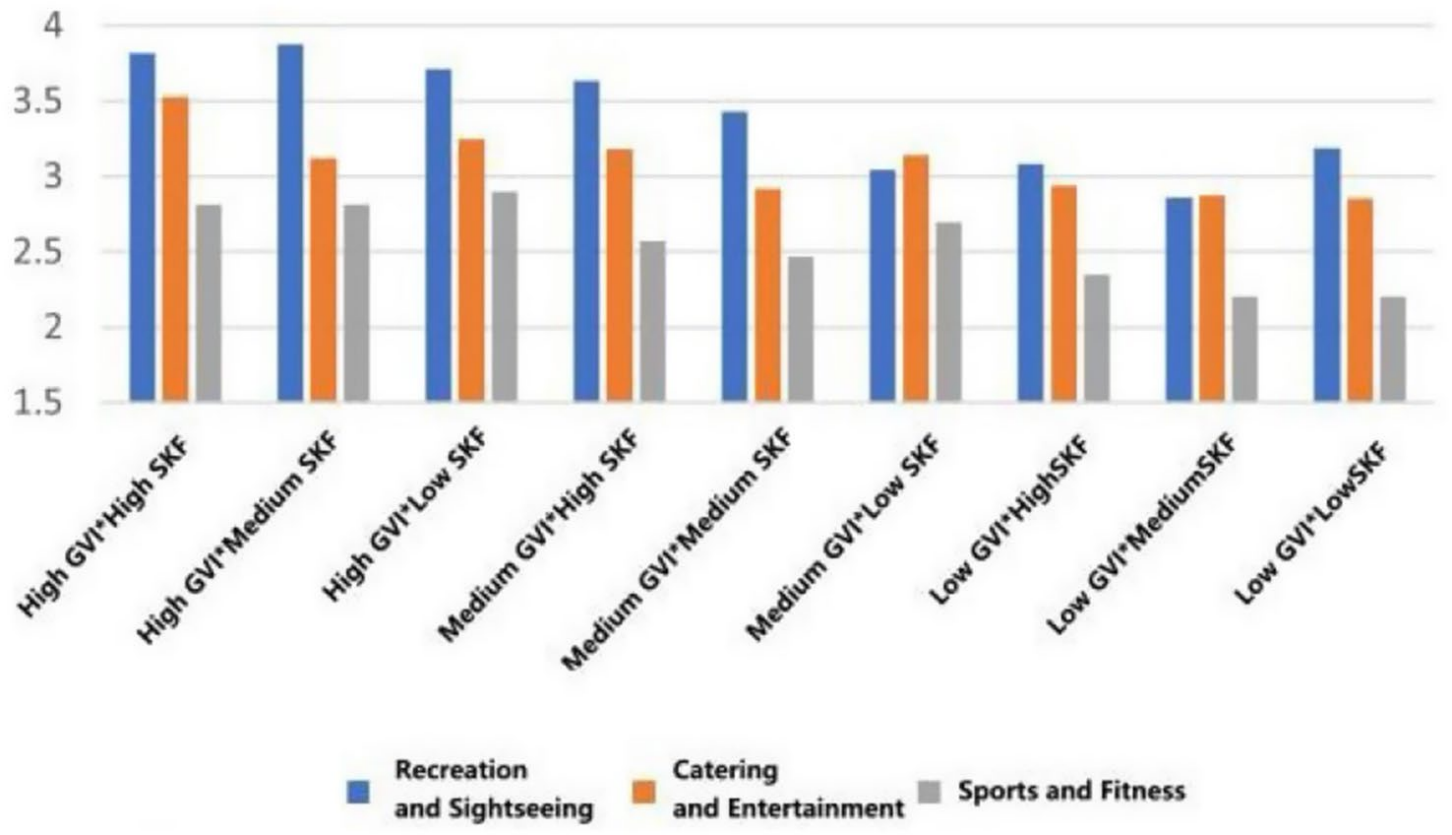
Mean changes in viewing under different environmental characteristics.

**Figure 17:**
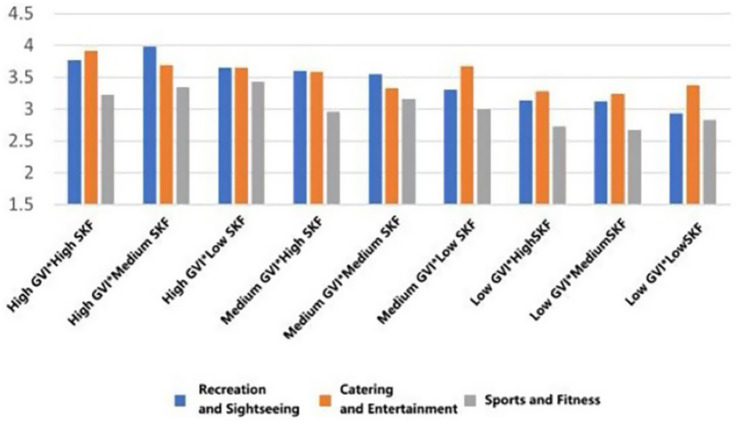
Mean changes in conversation under different environmental characteristics.

**Figure 18:**
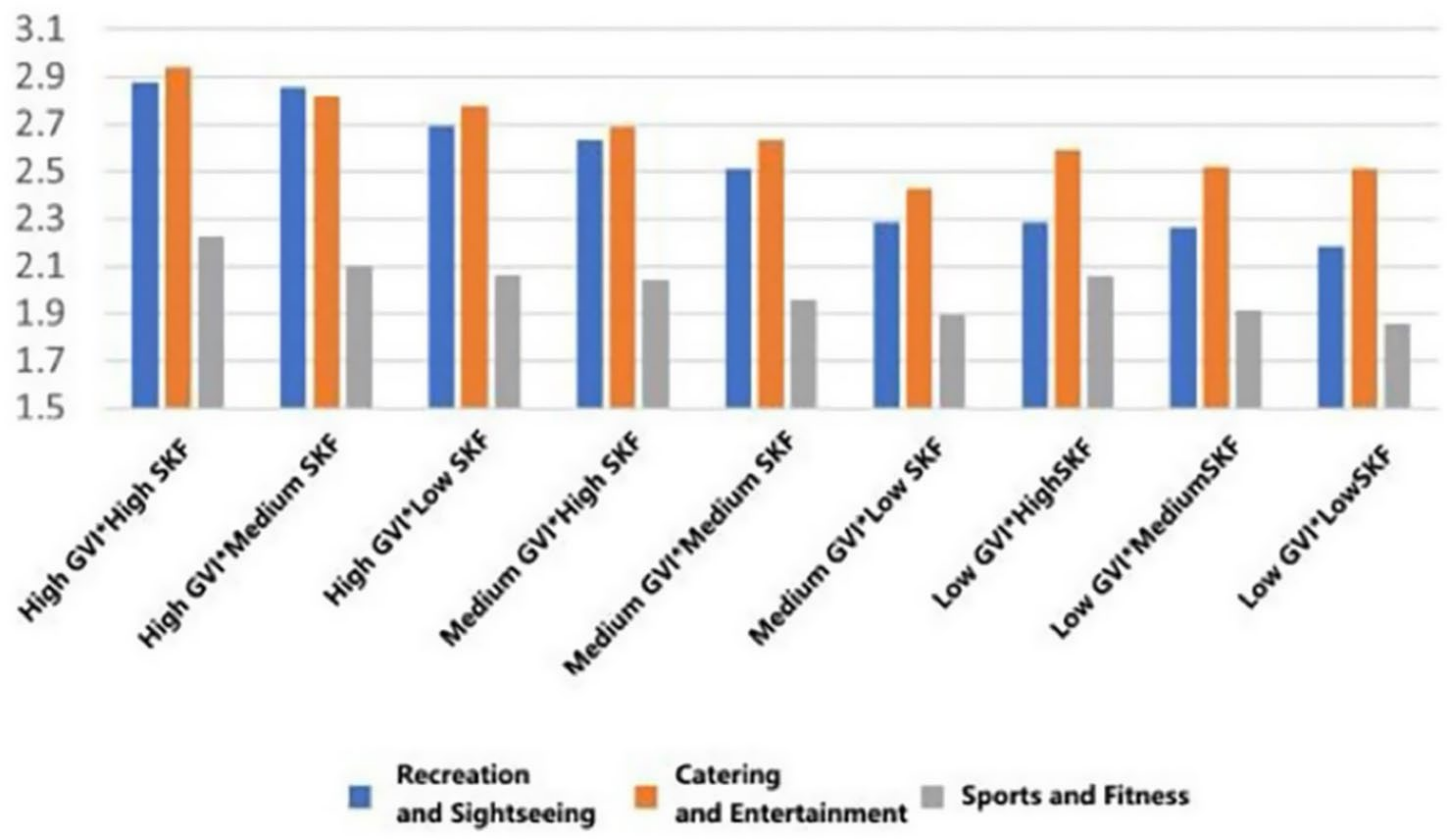
Mean changes in reading under different environmental characteristics.

Mean viewing willingness culminated in recreational rooftops featuring high GVI and medium SVF (*M* = 3.880, SD = 0.857), a configuration that appears to balance visual richness with a framed view. For conversation, the highest mean conditions were determined according to function: high GVI with medium SVF in recreational type (*M* = 4.004, SD = 0.755), high GVI with high SVF in entertainment areas (*M* = 3.832, SD = 0.739), and high GVI with low SVF in fitness spaces (*M* = 3.461, SD = 0.967). Lastly, the mean willingness to read was also maximum with high GVI and high SVF across all three rooftop types (*M* = 2.870, SD = 1.031; *M* = 2.990, SD = 0.710; *M* = 2.232, SD = 0.922), which is a good indicator of the broad appeal of bright, green environments in quiet, reflective activities (Figures 16–18).

#### 3.1.3 Social health effects

**Figure 19:**
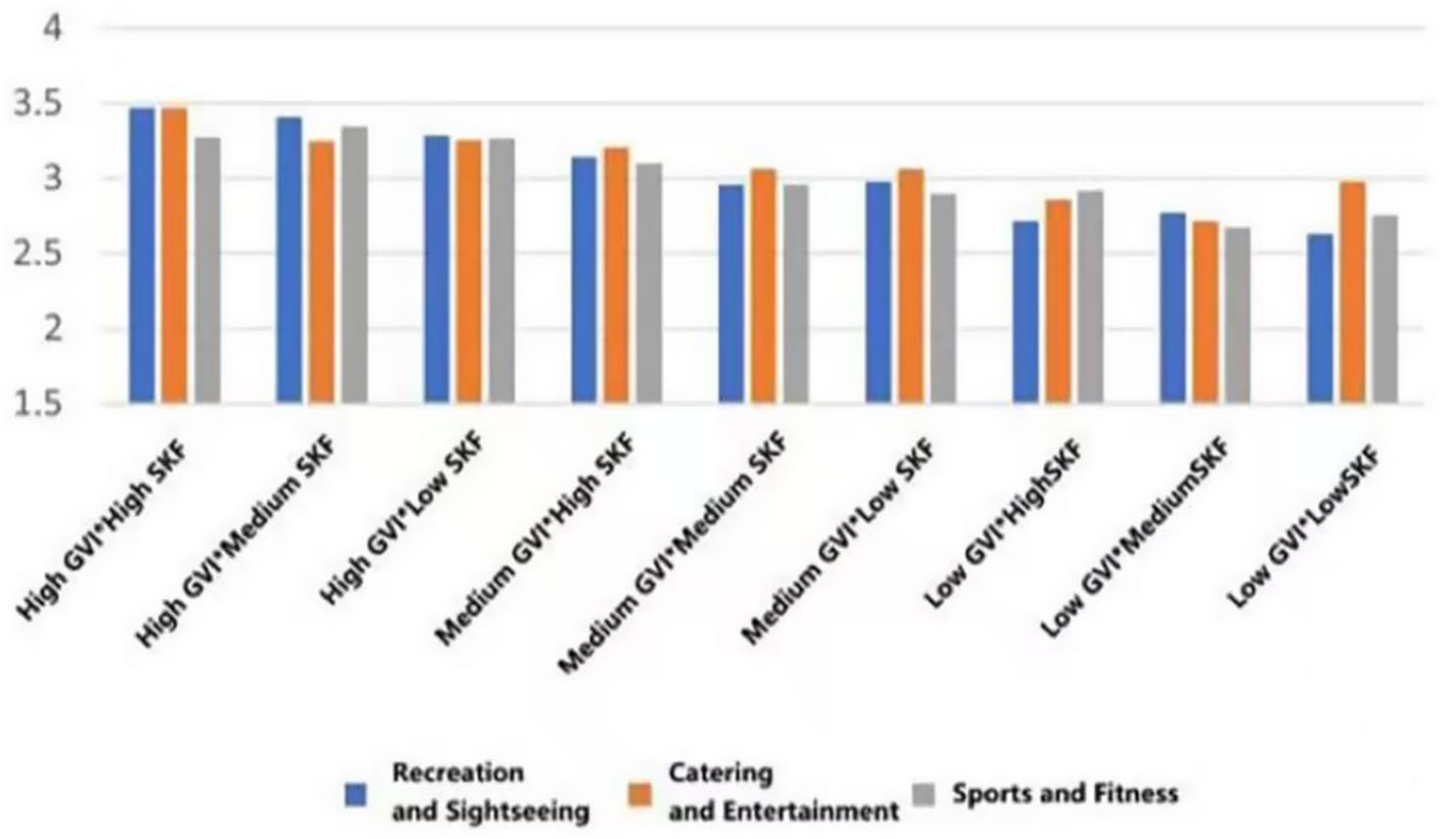
Mean changes in belonging under different environmental characteristics.

**Figure 20:**
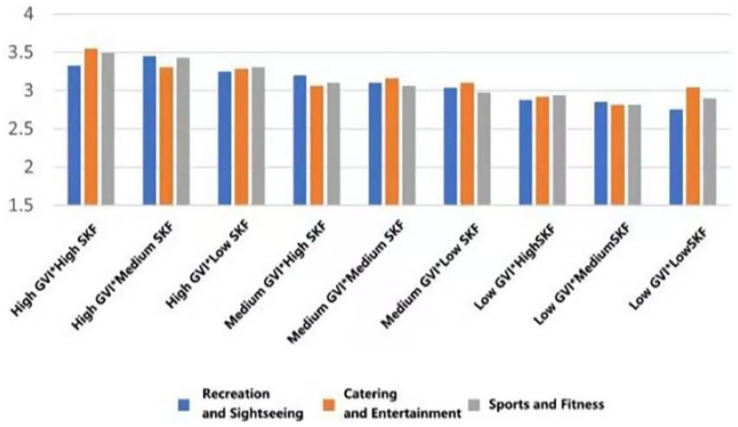
Mean changes in supporting under different environmental characteristics.

**Figure 21:**
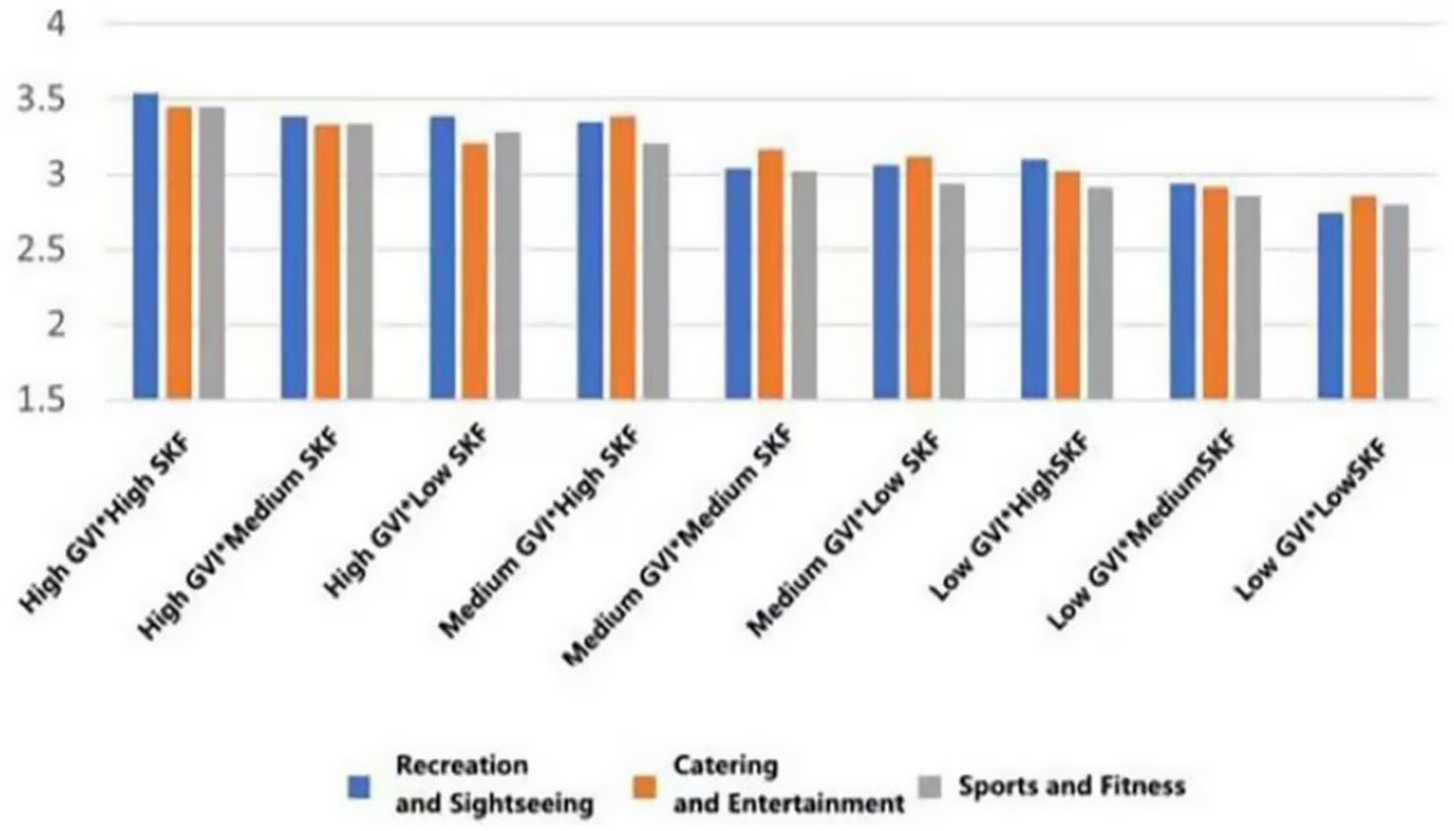
Mean changes in trust under different environmental characteristics.

**Figure 22:**
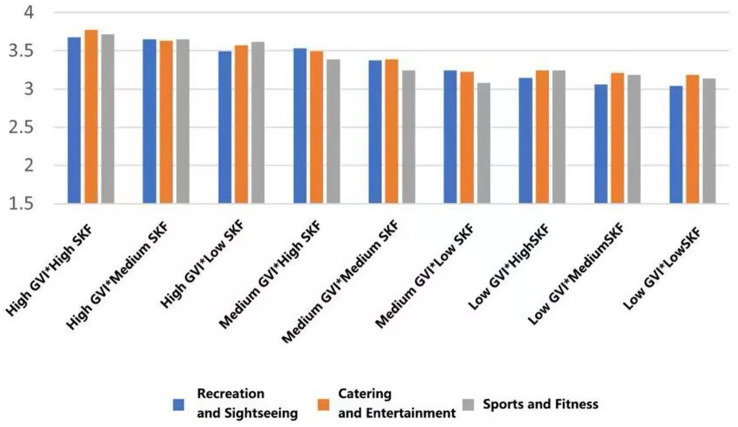
Mean changes in pleasure under different environmental characteristics.

**Figure 23:**
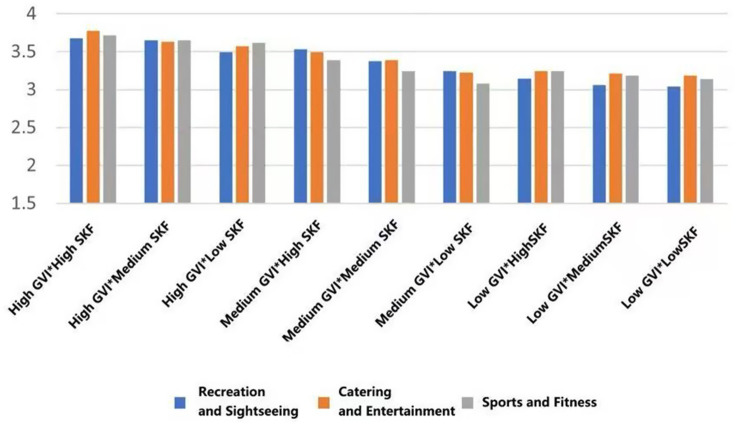
Mean changes in safety under different environmental characteristics.

In each functional typology, the mean ratings to the greatest extent were systematically measured in those settings that had both a high Green View Index (GVI) and a high Sky View Factor (SVF) (Belonging: *M* = 3.428, SD = 0.799; Support: *M* = 3.4465, SD = 0.834; Trust: *M* = 3.488, SD = 0.776; Pleasure: *M* = 3.692, SD = 0.776; Safety: *M* = 3.341, SD = 0.848); On the other hand, the lowest scores were consistent across the low GVI and low SVF condition (Belonging: *M* = 2.711, SD = 0.810; Support: *M* = 2.862, SD = 0.746; Trust: *M* = 2.819, SD = 0.622, Pleasure: *M* = 3.233, SD = 0.771; Safety: *M* = 2.694, SD = 0.751) (Figures 19–23).

### 3.2 Association between environmental variables and health indicators

To determine the independent relationship between the three major environmental predictors: Green View Index (GVI), Sky View Factor (SVF), and functional type and the multidimensional health outcomes, we employed Pearson correlation (for continuous GVI and SVF) and one-way ANOVA (for categorical functional type). Statistically significant associations (*p* < 0.05) informed subsequent multiple linear regression models, which quantified the combined influence of these variables.

#### 3.2.1 Association between GVI and health indicators

Green View Index demonstrated significant associations across all health dimensions. Regarding psychological health, it was negatively related to “Attention” (*r* = −0.40, *p* < 0.05) and “Excitement” (*r* = −0.40, *p* < 0.05), and positively related to “Relaxation” (*r* = 0.50, *p* < 0.01) (Supplementary Figure S1); Concerning the willingness to physical activity, GVI positively correlated with “Walking” (*r* = 0.870, *p* < 0.01), “Viewing” (*r* = 0.580, *p* < 0.01), “Conversing” (*r* = 0.700, *p* < 0.01) and “Reading” (*r* = 0.431, *p* < 0.05) (Supplementary Figure S2); Most significantly, GVI significantly correlated with all five social health indicators, including “Belonging,” “Support,” “Trust,” “Pleasure,” and “Safety” (*r* = 0.919, 0.923, 0.860, 0.887, 0.846, all *p* < 0.01) (Supplementary Figure S3).

In regression models controlling for SVF and functional type, GVI proved to be a general and significant predictor. The model predicting ‘Attention’ was significant [*F*(3, 93) = 3.98, *p* = 0.014, *R*^2^ = 0.42], with GVI showing a negative effect (*β* = −0.006, *p* = 0.023). Similarly, the model for ‘Excitement’ was significant [*F*(3, 93) = 12.20, *p* < 0.001, *R*^2^ = 0.58], with GVI as a negative predictor (β = −0.009, *p* = 0.008). Conversely, GVI was a positive predictor in the significant model for ‘Relaxation’ [*F*(3, 93) = 12.20, *p* < 0.001, *R*^2^ = 0.63; *β* = 0.008, *p* < 0.001]; In the domain of activity willingness, GVI was a strong positive predictor in significant models for ‘Walking’ [*F*(3, 93) = 77.44, *p* < 0.001, *R*^2^ = 0.92; *β* = 0.229, *p* < 0.001], ‘Viewing’ [*F*(3, 93) = 56.36, *p* < 0.001, *R*^2^ = 0.90; *β* = 0.294, *p* < 0.001], ‘Conversing’ [*F*(3, 93) = 42.94, *p* < 0.001, *R*^2^ = 0.87; *β* = 0.295, *p* < 0.001], and ‘Reading’ [*F*(3, 93) = 40.81, *p* < 0.001, *R*^2^ = 0.75; *β* = 0.175, *p* < 0.001]; GVI was the strongest and most consistent variable in the social domain. It was a significant positive predictor in models for ‘Belonging’ [*F*(3, 93) = 47.31, *p* < 0.001, *R*^2^ = 0.88; *β* = 0.277, *p* < 0.001], ‘Support’ [*F*(3, 93) = 44.15, *p* < 0.001, *R*^2^ = 0.87; *β* = 0.249, *p* < 0.001], ‘Trust’ [*F*(3, 93) = 74.87, *p* < 0.001, *R*^2^ = 0.92; *β* = 0.234, *p* < 0.001], ‘Pleasure’ [*F*(3, 93) = 52.17, *p* < 0.001, *R*^2^ = 0.89; *β* = 0.241, *p* < 0.001], and ‘Safety’ [*F*(3, 93) = 25.06, *p* < 0.001, *R*^2^ = 0.79; *β* = 0.272, *p* < 0.001] (Table 2).

**Table 2:** Regression analysis of environmental variables and health indicators.

Dependent variable	Independent variable							
	Green view rate		Sky view factor		Functional type			
					Catering and entertainment		Sports and fitness	
	Regression coefficient	Significance	Regression coefficient	Significance	Regression coefficient	Significance	Regression coefficient	Significance
Attention	−0.006*	0.023	−0.005*	0.035	×	×	×	×
Excitement	−0.009**	0.008	×	×	−0.026**	0.001	−0.022**	0.002
Relaxation	0.008**	0.000	0.008**	0.000	−0.011**	0.007	−0.011**	0.007
Stress	×	×	×	×	×	×	×	×
Engagement	×	×	−0.007**	0.001	×	×	×	×
Interest	×	×	×	×	×	×	−0.016**	0.000
Walking	0.229**	0.000	0.106**	0.000	×	×	−0.073*	0.020
Exercise	0.122**	0.000	×	×	0.181**	0.000	1.273**	0.000
Eating and drinking	0.175**	0.000	0.081**	0.004	0.649**	0.000	−0.327**	0.000
Viewing	0.294**	0.000	×	×	−0.314**	0.000	−0.846**	0.000
Conversing	0.295**	0.000	×	×	×	×	−0.411**	0.000
Reading	0.175**	0.000	×	×	0.146**	0.003	−0.498**	0.000
Belonging	0.277**	0.000	0.058*	0.011	×	×	×	×
Support	0.249**	0.000	0.046*	0.026	×	×	×	×
Trust	0.234**	0.000	0.112**	0.000	×	×	−0.083*	0.012
Pleasure	0.241**	0.000	0.089**	0.000	×	×	×	×
Safety	0.272**	0.000	×	×	×	×	−0.157*	0.013

The values in parentheses are *t*-values; *indicates *p* < 0.1, **indicates *p* < 0.05, ***indicates *p* < 0.001, X indicates *p* > 0.05; The analysis involved testing associations across multiple related health outcomes. While this increases the exploratory family-wise Type I error rate, the primary interpretation emphasizes the magnitude of standardized effects (β) and the **variance explained (*R*^2^)**. The analysis involved testing associations across multiple related health outcomes. While this increases the exploratory family-wise Type I error rate, our interpretation emphasizes the consistency of patterns across related measures, the magnitude of effect sizes (e.g., correlation coefficients r, standardized beta coefficients β), and the theoretical plausibility of the findings, rather than relying solely on isolated *p*-values.

#### 3.2.2 Association between SVF and health indicators

It was found to be psychologically negatively related to Engagement (*r* = −0.61, *p* < 0.01) and Attention demand (*r* = −0.36, p < 0.05), and positively to relaxation for SVF (*r* = 0.52, *p* < 0.01) (Supplementary Figure S4). It had a weak correlation with behavioral intentions for physical activity with the only significant positive correlating variable of walking intention (*r* = 0.402, *p* < 0.05) (Supplementary Figure S5); For social health, SVF correlated positively with Trust (*r* = 0.411, *p* < 0.05) and Pleasure (*r* = 0.892, *p* < 0.01), but not with Belonging, Support, or Safety (Supplementary Figure S6).

Regression equations indicated that SVF was associated with lower attention. The model for ‘Attention’ was significant [*F*(3, 93) = 3.98, *p* = 0.014, *R*^2^ = 0.42], with SVF as a negative predictor (*β* = −0.005, *p* < 0.05) and was the sole significant predictor for reduced engagement [*F*(3,93) = 5.09, *p* = 0.005, *R*^2^ = 0.48; *β* = −0.007, *p* < 0.01]. It independently promoted relaxation [*F*(3, 93) = 12.20, *p* < 0.001, *R*^2^ = 0.63; *β* = 0.008, *p* < 0.01]. Regarding activity willingness, SVF was a significant positive predictor in the model for walking [*F*(3, 93) = 77.44, *p* < 0.001, *R*^2^ = 0.92; *β* = 0.106, *p* < 0.001]. In social outcomes, SVF increased perceived trust [*F*(3, 93) = 74.87, *p* < 0.001, *R*^2^ = 0.92; *β* = 0.112, *p* < 0.001] and pleasure [*F*(3, 93) = 52.17, *p* < 0.001, *R*^2^ = 0.89; *β* = 0.089, *p* < 0.001] in the combined models (Table 2).

#### 3.2.3 Association between functional type and health indicators

The effect of functional type as a discrete variable was estimated with ANOVA. It produced meaningful inter-group disparities among the psychological measures of the variables of “Excitement” (*p* < 0.01) and “Interest” (*p* < 0.001) (see Supplementary Table S10). In the case of physical activity willingness, fundamental differences were observed by profile with significant disparity in scores of “Exercise,” “Eating and drinking”, “Viewing”, “Conversation”, and “Reading” (all *p* < 0.01) (see Supplementary Table S11). Conversely, functional type moderated indistinctly the effects on any social health indicator across various types (*p* > 0.05) (see Supplementary Table S12).

When fit as a dummy variable (recreation and sightseeing as the baseline), catering/entertainment and sports/fitness areas were found to be less exciting [*F*(3, 93) = 3.98, *p* < 0.001, *R*^2^ = 0.58] and relaxing [*F*(3, 93) = 12.20, *p* < 0.001, *R*^2^ = 0.63] than recreational areas (for ‘Excitement’: *β* = −0.025 and −0.029, *p* < 0.01; for ‘Relaxation’: *β* = −0.011 in both cases, *p* < 0.01). Interest was also significantly diminished by the use of fitness spaces [*F*(3, 93) = 21.21,*p* < 0.001, *R*^2^ = 0.76;β = −0.016, *p* = 0.001]; In the domain of activity willingness, sports/fitness spaces were a strong positive predictor in the model for exercise intention [*F*(3, 93) = 267.07, *p* < 0.001, *R*^2^ = 0.90; *β* = 1.273, *p* < 0.001] but showed negative coefficients in models for eating/drinking [*F*(3, 93) = 267.07, *p* < 0.001, *R*^2^ = 0.90; *β* = −0.327, *p* < 0.001], sightseeing [*F*(3, 93) = 56.36, *p* < 0.001, *R*^2^ = 0.90; *β* = −0.846, *p* < 0.001], conversing [*F*(3, 93) = 42.94, *p* < 0.001, *R*^2^ = 0.87; *β* = −0.411, *p* < 0.0001], and reading [*F*(3,93) = 40.81, *p* < 0.001, *R*^2^ = 0.75;*β* = −0.498, *p* < 0.001]. Catering/entertainment spaces were a positive predictor in the model for eating and drinking intention [*F*(3, 93) = 111.24, *p* < 0.001; *β* = 0.649, *p* < 0.001] but a negative predictor in the model for sightseeing willingness [*F*(3, 93) = 56.36, *p* < 0.001; *β* = −0.314, *p* < 0.001]; In social health models, functional type had limited influence. Sports/fitness spaces showed a small negative coefficient in the models for trust [*F*(3, 93) = 74.87, *p* < 0.001, *R*^2^ = 0.92; *β* = −0.083, *p* < 0.05] and safety [*F*(3, 93) = 25.06, *p* < 0.001, *R*^2^ = 0.79; *β* = 0.157, *p* < 0.05] (Table 2).

### 3.3 Interaction analysis between environmental variables and health indicators

To quantify the influence of design elements, a combined Effects model was established. The independent variables were coded as dummy variables: “Recreation and Sightseeing” function, “low Green View Index (GVI)”, and “low Sky View Factor (SVF)” were set as the reference baseline. Dummy variables were formed that indicated the types of functional (entertainment, fitness), GPI (medium, high), and SVF (medium, high). Three groups of terms of interaction, each term indicating a type of functional and GVI, functional type and SVF, and GVI and SVF, were entered into the model simultaneously to test the synergistic effect of the elements. This specification of the model enables the regression coefficients to be directly interpreted as the net incremental health benefit of other combinations of factors relative to the baseline case (see Supplementary Table S13). The overall model explained 46.8% of the variance in psychological health (R^2^ = 0.468), 50.3% in physical willingness (R^2^ = 0.503), and 46.3% in social health (R^2^ = 0.463).

**Figure 24:**
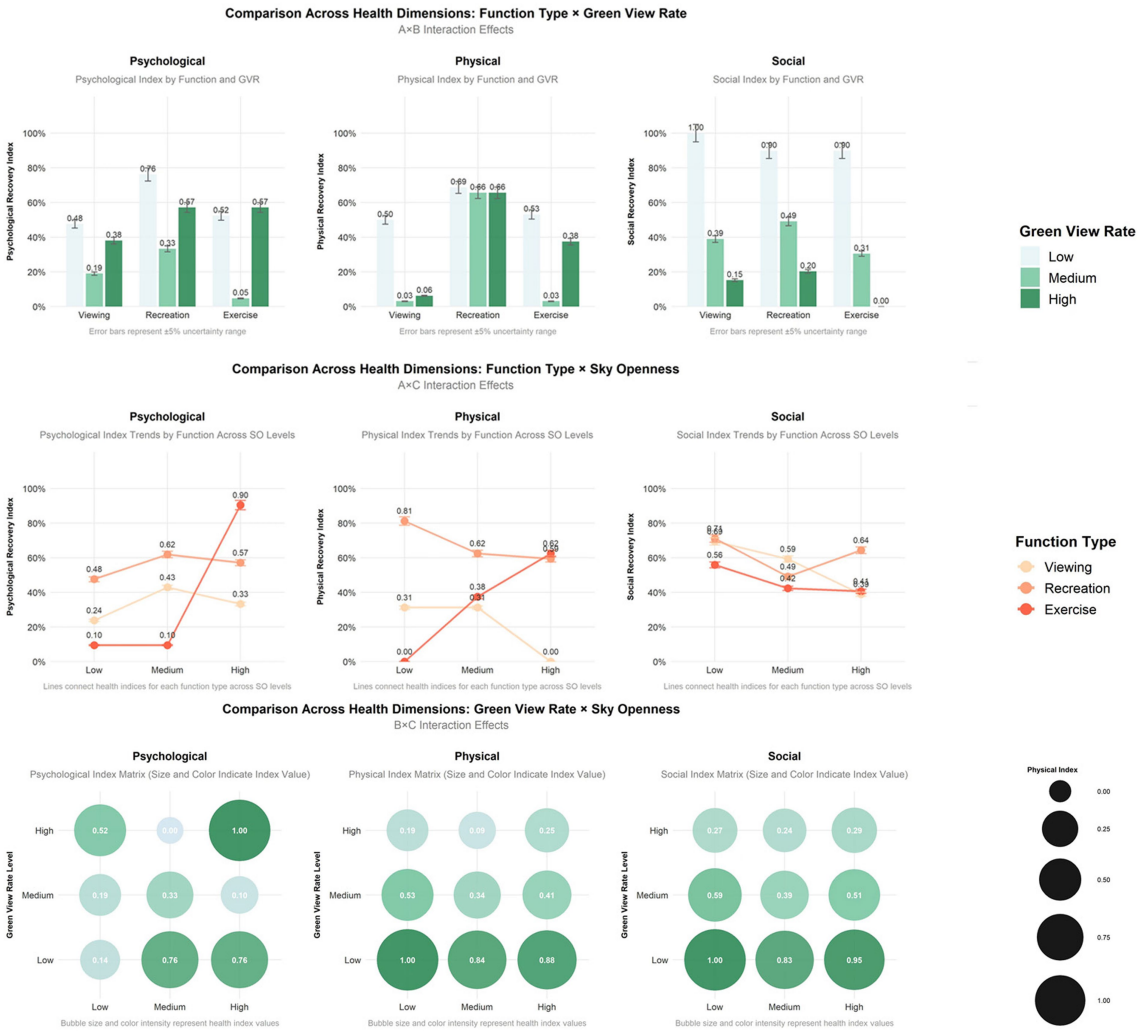
Analysis of the interaction effects of environmental characteristics.

As can be seen in Figure 24, the restorative effect of GVI showed a nonlinear differentiation as the level increases. Medium GVI provided a background profit to psychological (*β* = 0.005, *p* < 0.1), physical (*β* = 0.294, *p* < 0.05), and social health (*β* = 0.291, *p* < 0.001). Nevertheless, at the high level of GVI, the positive influence on the physical (*β* = 0.504, *p* < 0.001) and social health was further increased (*β* = 0.572, *p* < 0.001), whereas the principal impact on the psychological health was non-significant. In comparison, the health tenets of SVF showed a strong linear trend of improvement with an increase in levels. The stepwise transition of medium to high SVF was consistently and significantly fostering psychological (*β* = 0.019, *β* = 0.107, *p* < 0.05), physical (*β* = 0.081, *β* = 0.089, *p* < 0.05) and social health (*β* = 0.095, *β* = 0.112, both *p* < 0.001); Fitness-oriented spaces promoted physical health (*β* = 0.088, *p* < 0.05) while exerting a minor negative effect on psychological health (*β* = −0.004, *p* < 0.05); entertainment rooftop space, conversely, showed an independent positive contribution to psychological health (*β* = 0.012, *p* < 0.05).

Secondly, the combination of entertainment rooftop space and high GVI produced the strongest interaction effect. Their term of interaction had a positive significant consequence on the psychological (*β* = 0.207, *p* < 0.05), physical (*β* = 0.167, *p* < 0.1), and social health (*β* = 0.118, *p* < 0.1). The interaction effect between fitness spaces and high GVI was relatively limited, showing only weak positive contributions to physical (*β* = 0.069, *p* < 0.1) and social health (*β* = 0.042, *p* < 0.1). Notably, the interaction between fitness spaces and high SVF yielded the highest effect for physiological health restoration (*β* = 0.149, *p* < 0.05) and also produced a significant positive moderating effect on psychological health recovery (*β* = 0.043, *p* < 0.05).

Furthermore, the “high GVI and high SVF” combination exhibited significant positive interactive effects on the restoration of psychological (*β* = 0.004, *p* < 0.05), physical (*β* = 0.039, *p* < 0.05), and social health (*β* = 0.074, *p* < 0.05). Notably, the “medium GVI and high SVF” combination also independently and significantly promoted psychological health (*β* = 0.048, *p* < 0.05), and its interaction coefficient was greater than that of the “high GVI and high SVF” combination (*β* = 0.048 > 0.004, *p* < 0.05).

## 4 Discussion

### 4.1 Effects of rooftop environmental indicators on health benefits

This paper elucidates the associations between environmental exposure (Green View Index, GVI; Sky View Factor, SVF; functional type) and multidimensional restorativeness (psychological, physical, and social health) in rooftop greening areas.

Descriptive analysis revealed that the distribution of the health benefits is not homogeneous but depends on the functional dependency and specificity of the environmental conditions. In the case of psychological health, the predominant recovery pathways were different in functional space. Recreation and Sightseeing space showed complex psychological recovery benefits, with the greatest response being towards relaxation and interest, and the lowest response to cognitive pressure in situations of high or medium GVI, high SVF combination. This is consistent with Attention Restoration Theory, which posits that this kind of low-demand, high-naturalness environment is effective at decomposing the psyche (Tennessen and Cimprich, 1995). Fitness-oriented spaces, on the contrary, were characterized by a more complicated psychological profile. They exhibited reduced recovery scores (e.g., excitement and interest) and a small net negative response to psychological state in main-effect models, which indicates a possible conflict between the arousal that physical activity goals entail and the low-arousal state that psychological relaxation would imply. Nevertheless, within such spaces, superior visual surroundings still contributed positively to psychological recovery, indicating that environmental quality can provide a cumulative benefit alongside, rather than solely counteracting, the activity’s primary demands. This is consistent with the results of Han (2010) in ground-level parks, where nature coupled with light exercise maximized mood and attention.

The correlation between the behavioral Intentions for various types of activities and the environmental factors was more direct in the dimension of physical health (activity willingness). Willingness was always the highest in the conditions of low GVI and high SVF, such as walking and reading, which is in line with the results that open grasslands facilitate leisure activity. Nevertheless, the readiness to exercise intensively was the highest in the case of the high GVI, medium-to-low SVF combination. This is partially opposite to the results obtained by Gatersleben and Andrews (2013) on forest trails- their study revealed that high openness and low refuge combinations provided an optimal result on the restorative perception. The current evidence indicates that, in the case of goal-oriented exercise, an average level of visual enclosure (lesser SVF) can be more favorable for paying attention to and decreasing external interference. This is an indication of environment preference redefined through functional goals.

In the case of social health, all indicators (belonging, trust, pleasure, etc.) consistently scored high in the high GVI, high SVF grouping. This demonstrates that the naturalness (GVI) and openness (SVF) are virtually sufficient factors in the perception of social health, and it is stronger than the impact of any functional type alone in the given setting, i.e., rooftops. This is probably intimately linked to the fact that rooftop spaces are controlled semi-public spaces. In comparison with the multifaceted safety issues which may be involved in ground-level parks, constructed rooftop gardens are often characterized by well-defined boundaries, controlled sightlines, and high levels of human maintenance (Fernandez-Cañero et al., 2013; Loder, 2014; Chen et al., 2022), which may mitigate safety concerns associated with dense vegetation in ground-level settings (Hunter et al., 2019; Tsantopoulos et al., 2018; Mesimäki et al., 2017; Jungels et al., 2013).

Association and regression analyses consistently identified GVI as a broad and positive predictor across health dimensions. Its core role lies in providing natural contact and visual buffering (Qiu et al., 2013). The positive associations with all social health dimensions support the Biophilia hypothesis (Ulrich, 1993; van den Berg et al., 2010; Swanwick, 2009). Regarding activity willingness, the positive relationship of GVI with such activities as walking, conversing, and sightseeing shows that it facilitates spontaneous low-intensity activities through improving the attractiveness of an environment (Hunter et al., 2015; Pretty et al., 2005). Nevertheless, it had a nonlinear effect on psychological health. Although regression analysis indicated that GVI positively affected relaxation and had a negative effect on excitement, the interaction effect analysis also indicated that the primary effect of high GVI on psychological health became insignificant. This observation is in line with arguments about threshold effects of greenery (Jiang et al., 2014). Our data suggest that beyond a certain point, the potential restorative benefit of increased greenery may be offset by increased visual complexity, a balance that appears particularly sensitive in the constrained rooftop context. Conversely, ground-level green areas, complete with their micro-topographical buffers (by transitioning to a shrub layer), are capable of supporting psychological benefits at greater levels of greenery (35–40%) (Luo and Jiang, 2022). This, combined with structural design factors such as load-bearing capacity and waterproofing in rooftop designs, means that the Sky View Factor (SVF) plays a crucial role in improving the psychological decompression and efficacy in social perception as a way of reducing their feeling of oppression that is often felt in a high-density building (Kaplan, 2001; Hadi et al., 2018; Shooshtarian and Ridley, 2017). SVF contributed to psychological restoration by promoting relaxation and reducing perceived engagement, indicating a reduction in environmental demands and cognitive load (Mesimäki et al., 2019; Jungels et al., 2013). This regulatory role extended to the social domain, where increased SVF was associated with greater trust and pleasure, likely linked to the enhanced sense of control from open views (Lee et al., 2015; Chen et al., 2022; Fernandez-Cañero et al., 2013). Consistent with this regulatory pathway, SVF’s influence on activity willingness was specific, primarily encouraging walking rather than broadly stimulating behavioral intentions for physical activity.

Functional typology emerged as a primary organizing variable for health outcomes. Both ANOVA and regression analyses confirmed it generated the most significant between-group differences, particularly for activity willingness (e.g., exercise). Such a finding highlights the significance of the matching of supply–demand: certain functions, with the help of material cues, explicitly convey the nature of the activities, which they sustain, thus strongly predetermining the behavioral intention and psychological expectations of the users (Zeng et al., 2019; Veen et al., 2016). As an example, catering areas contributed much to decreasing excitement and increasing interest (Smith, 2015; Zacher et al., 2014; Nordh and Østby, 2013), whereas fitness areas contributed much to increasing willingness to exercise and decreasing relaxation (Heyn et al., 2004; Pretty et al., 2005). It can be viewed in terms of the environmental demand theory: the very concept of fitness facilities means that there should be anticipations of physical activity and increased arousal, which can become incompatible with the aim of mental restoration after mental fatigue (Mikkelsen et al., 2017; Barton and Pretty, 2010). On the other hand, sightseeing roofspace is a low-monitor situation that facilitates easy attention and mental release. Nevertheless, the influence of functional type on the social health indicators was not strong, which makes it possible to suggest that positive social experiences depend more on underlying environmental quality than on explicit functional labels.

Finally, the interaction analysis revealed that maximizing health benefits relies not on optimizing single elements in isolation, but on their strategic combination, aligning with the cumulative synergy model (Deng et al., 2020). For instance, the interplay between GVI and SVF was crucial for balancing visual complexity and openness While high GVI alone showed limited psychological benefits, the combination of ‘high GVI’ and ‘high SVF’ yielded substantial positive interaction between the two variables in all the dimensions of health. What is more essential is the fact that the synergy between medium GVI and high SVF on psychological health was even higher. It means that in the framework of rooftop areas that emphasize the psychological recovery, a mechanism of moderately greenery with high SVF can be better than an extremely high one. This mix offers enough natural elements and does not create the possible feeling of oppression by the excessively dense vegetation. The principle behind this is related to the conclusions drawn in the studies of the green space at the ground level, which highlights the importance of edges and visual permeability, though the limits may vary depending on the nature of the particular space at the rooftop due to the presence of the stronger sense of being enclosed (Hauru et al., 2012; Rall et al., 2017). Significant interaction terms demonstrate that the visual environment can be used to enhance or change the health output of certain functions. As an example, the strong interactive effect on psychological health when combining ‘catering and entertainment spaces’ and, with high GVI, indicates that in an environment which has the inherent properties of social arousal, a high-greenery natural environment will not lead to interference. Rather, it probably offers a visual light background that cushions the possible cognitive load of social activities and thus attains the effect of additions of social pleasure and natural restorativeness. Conversely, the interaction of the two variables of fitness spaces and high SVF, can serve as a compensatory solution to the psychological stress inherent in exercise spaces, where an open vista can allow the practitioner to recover the calm more rapidly when taking a rest period (Jiaxin, 2023; Luo and Jiang, 2022; Shooshtarian and Ridley, 2017). This suggests the possibility to harmonize various health goals in a single-function environment through the interactive design of the environmental factors.

### 4.2 Strengths and limitations

The principal strengths of this research are threefold. First, it presents a methodologically sound system of evaluating the rooftop green spaces, which is novel in combining the controlled exposure to VR with the neurophysiological-derived indices (EEG) measures and multidimensional subjective reporting. The triangulation can be used to accurately decompose the independent and interactive effects of design variables that are of interest. Second, it makes an important theoretical contribution to the literature of the restorative environment. Stepping out of generic prescriptions, it determines the context-dependent operations and nonlinear thresholds of the vertical, confined space of rooftops, and codifies the leading role of visual permeability (the GVI-SVF balance). Third, it provides a nuanced evidence base to inform design. The quantified interaction effects move past simplistic “more green is better” mandates, offering insights for tailoring spaces toward primary goals such as cognitive respite, social vitality, or physical activation.

Several limitations of this study should be acknowledged. First, regarding the analytical approach, the two-stage procedure (correlation/ANOVA screening followed by regression) employed to explore fine-grained associations reflects a pragmatic strategy within the linear modeling framework. A fundamental constraint lies in the simplifying assumptions of linear regression itself, which, while clarifying main and interactive effects, may not fully capture more complex, non-linear human-environment relationships. In addition, the multiple statistical comparisons conducted across related outcome dimensions pose an inherent limitation regarding the inflation of Type I error. Future research could extend these findings by adopting non-linear modeling techniques better suited to theorizing and testing such complex pathways, alongside confirmatory designs with appropriate statistical corrections. Second, the participant sample, comprised predominantly of adult, may not fully represent the broader urban population demographics, which could limit the generalizability of the preference and perception outcomes. Third, although the VR-based methodology offered the necessary level of experimental control, it had to ignore multisensory preconditions (e.g., wind, temperature, ambient sound) and long-term behavioral patterns that are inherent to real-life conditions. The findings should be confirmed by future studies using field studies that incorporate the use of biometric sensors and consider the moderating factor of the microclimate. Finally, the cross-sectional design of the experiment precludes causal inferences regarding long-term health outcomes. Moreover, while the experimental design controlled exposure, the identified statistical relationships remain associational in nature. Future longitudinal or experimental studies with repeated measures are needed to establish temporal precedence and strengthen causal inference regarding the restorative effects of these environmental configurations.

## 5 Conclusion

This study systematically examined the restorative effects of open rooftop greening spaces by addressing three targeted research questions.

The distribution nature of health indicators provided evidence of a landscape of functional and environmental particularity. Although high visual quality invariably correlated with superior psychological and social outcomes in terms of high GVI and SVF, willingness to engage in physical activity showed specific calibration, and optimum conditions were dependent on the type of activity (e.g., high-intensity exercise favored fitness spaces with low SVF).

The associations of key environmental variables with health outcomes delineated distinct roles. GVI was the broadest predictor, foundational for social well-being and light activities. The effect of SVF was the major mediator of psychological decompression and particular social feelings. Functional type served as the major filter with a significant effect on the intent of activities and the affective involvement, and little influence on social health independently.

The interactive effects among variables provided evidence of the significance of the synergistic combination, and not the maximization, for the best results. The fact that the model of medium GVI/high SVF performs better than the model of high GVI/high SVF in psychological recovery provides an important design enhancement. Significant functional-visual interactions further indicate that greenery has the potential to enhance the salutary effects of social space, whereas openness can alleviate pressure in exercise space.

To sum up, this study establishes rooftop greening as an essential element of salutary city space. It is context sensitive in its effectiveness, and functional-visual synergy is highly significant. The results form an empirical basis to eliminate generic greening requirements to the precision engineering of rooftop environments, enabling the creation of vertical oases that effectively promote mental restoration, social connection, and physical vitality within the high-density urban fabric.

## Data Availability

The original contributions presented in the study are included in the article/supplementary material, further inquiries can be directed to the corresponding author/s.
