# Supplementary material for: Assessing the restorative benefits of open rooftop green spaces characteristics using virtual reality and EEG analysis
Source: Front Psychol. 2026 May 20;17:1807425. doi: 10.3389/fpsyg.2026.1807425 (PMC13229816; doi:10.3389/fpsyg.2026.1807425)
Supplement: Supplementary file 1 [file Supplementary_file_1.docx]

Table S1 Sample of Open Roof Greening Space in Chengdu

| sample | Name | sample | Name |
| --- | --- | --- | --- |
| Sample1 | IFS International Finance Center | sample15 | Wangfujing Discovery |
| Sample2 | Guangqun Plaza | sample16 | Intime Center in99 |
| Sample3 | ICD Mall | sample17 | Honghui Plaza |
| Sample4 | Maoye Renhe Springren East Store | sample18 | Renhe New Town |
| Sample5 | Pengruili Qingyang Plaza | sample19 | Tencent Building |
| Sample6 | Fortune Mall | sample20 | Southern Tianfu Plaza |
| sample7 | Longhu Xichen Tianjie | sample21 | Three Gorges Corporation Building |
| sample8 | Raffles City Plaza | sample22 | MKL Lifestyle Aesthetics Center |
| sample9 | Ox Square | sample23 | High-tech Chuangzhi Plaza |
| sample10 | Youfang Shopping center | sample24 | China Construction Binhu Design Headquarters |
| sample11 | Merchant Magic Cube | sample25 | Beijing University of Aeronautics and Astronautics Chengdu Research Institute |
| sample12 | Electric Power Research Institute | sample26 | Haitian Group plaza |
| sample13 | China Resources Mixc plaza | sample27 | Power China building |
| sample14 | InCity | sample28 | China Telecom building |

Table S2 Classification Standards for Green View Rate of Open Roof Greening Spaces

| Classification Standards | Numerical range(%) | Description |
| --- | --- | --- |
| Level1 | 0-0.05 | There is almost no greenery in sight, and you can hardly sense any greenery. |
| Level2 | 0.05-0.15 | There is very little greenery in sight, but it can be faintly perceived. |
| Level3 | 0.15-0.30 | There is a small amount of greenery in view, and the greenery can be perceived. |
| Level4 | 0.30-0.50 | There is a lot of greenery in view, and the greenery is clearly noticeable. |
| Level5 | 0.50-0.65 | The view is filled with greenery, allowing you to directly experience it. |
| Level6 | 0.65-1 | The main thing in view is greenery, and you can directly perceive it. |

Table S3 Classification Standards for Green View Rate of Samples in Chengdu

| sample  No | GVI | Level | sample  No | GVI | Level | sample  No | GVI | Level |
| --- | --- | --- | --- | --- | --- | --- | --- | --- |
| Sample1 | 31.88% | 4 | sample11 | 8.38% | 2 | sample20 | 9.38% | 2 |
| Sample2 | 35.88% | 4 | sample12 | 23.25% | 3 | sample21 | 20.38% | 3 |
| Sample3 | 16.88% | 3 | sample13 | 16.38% | 3 | sample22 | 31.75% | 4 |
| Sample4 | 9.13% | 2 | sample14 | 34.00% | 4 | sample23 | 9.75% | 2 |
| sample5 | 16.63% | 3 | sample15 | 13.00% | 2 | sample24 | 19.50% | 3 |
| sample6 | 40.00% | 4 | sample16 | 10.63% | 2 | sample25 | 17.88% | 3 |
| sample7 | 14.88% | 2 | sample17 | 33.88% | 4 | sample26 | 18.50% | 3 |
| sample8 | 9.88% | 2 | sample18 | 30.88% | 4 | sample27 | 32.13% | 4 |
| sample9 | 35.75% | 4 | sample19 | 24.00% | 3 | sample28 | 9.88% | 2 |
| sample10 | 25.00% | 3 |  |  |  |  |  |  |

Table S4 Classification Standards for Sky View Factor of Open Rooftop Greening Spaces

| Level | Numerical range(%) | Description |
| --- | --- | --- |
| Level1 | 0-0.01 | The sky is almost invisible within the field of view, and the openness of the space is extremely low. |
| Level2 | 0.01-0.10 | The sky occupies a smaller area in the field of view, and the sense of spatial openness is relatively low. |
| Level3 | 0.10-0.25 | The sky is fairly visible in the field of view, and the sense of openness is moderate. |
| Level4 | 0.25-0.40 | There is a larger area of sky in view, and the sense of openness is greater. |
| Level5 | 0.40-0.84 | The main thing in view is the sky, and the space is very open. |

Table S5 Classification Standards for Sky View Factor of Samples in Chengdu

| Sample  No | SKF | Level | Sample  No | SKF | Level | Sample  No | SKF | Level |
| --- | --- | --- | --- | --- | --- | --- | --- | --- |
| sample1 | 26.38% | 4 | sample11 | 18.13% | 3 | sample20 | 9.38% | 2 |
| sample2 | 8.50% | 2 | sample12 | 26.13% | 4 | sample21 | 18.63% | 3 |
| sample3 | 20.38% | 3 | sample13 | 35.63% | 4 | sample22 | 15.50% | 3 |
| sample4 | 21.13% | 3 | sample14 | 17.38% | 3 | sample23 | 13.88% | 3 |
| sample5 | 19.38% | 3 | sample15 | 25.88% | 4 | sample24 | 26.00% | 4 |
| sample6 | 25.50% | 4 | sample16 | 8.88% | 2 | sample25 | 27.38% | 4 |
| sample7 | 19.00% | 3 | sample17 | 10.00% | 2 | sample26 | 28.25% | 4 |
| sample8 | 10.50% | 3 | sample18 | 11.88% | 3 | sample27 | 18.13% | 3 |
| sample9 | 19.38% | 3 | sample19 | 26.13% | 4 | sample28 | 20.38% | 3 |
| sample10 | 17.88% | 3 |  |  |  |  |  |  |

Table S6 Classification Standards for Functional Type of Open Rooftop Greening Spaces

| sample  No | Functional type | sample  No | Functional type | sample  No | Functional type |
| --- | --- | --- | --- | --- | --- |
| sample1 | 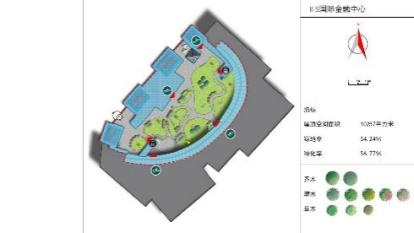  Catering and Entertainment | sample11 | 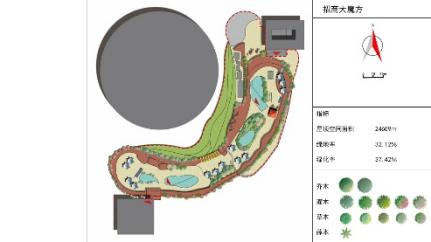  Recreation and Sightseeing | sample20 | 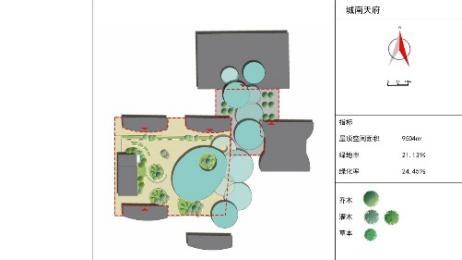  Catering and Entertainment |
| sample2 | 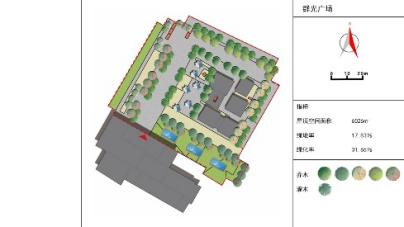  Recreation and Sightseeing | sample12 | 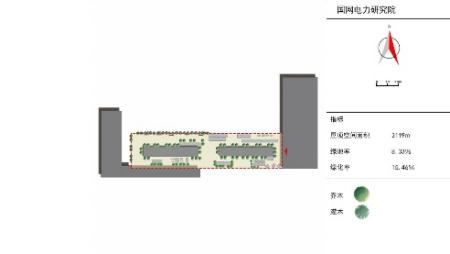  Catering and Entertainment | sample21 | 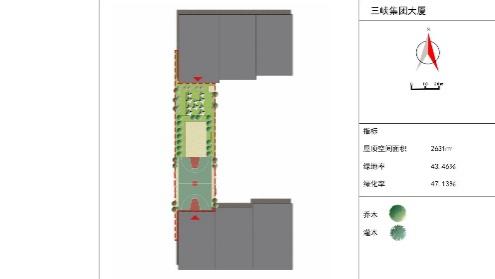  Recreation and Sightseeing |
| sample3 | 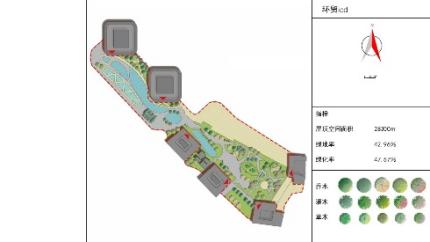  Catering and Entertainment | sample13 | 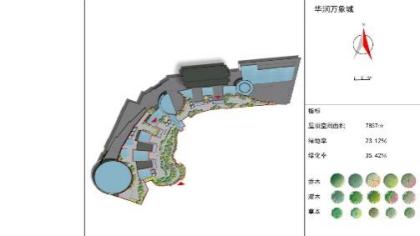  Recreation and Sightseeing | sample22 | 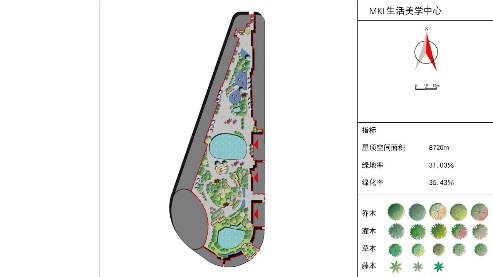  Recreation and Sightseeing |
| sample4 | 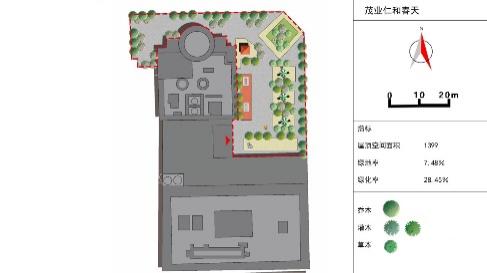  Sports and Fitness | sample14 | 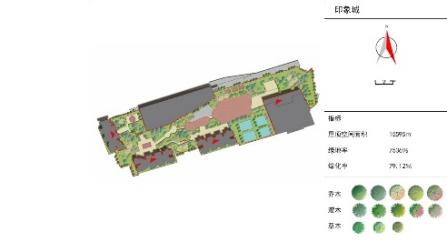  Catering and Entertainment | sample23 | 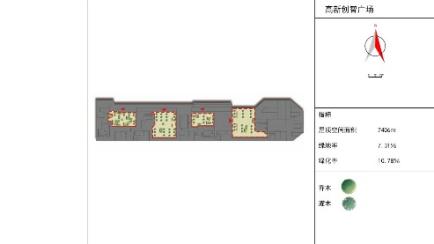  Catering and Entertainment |
| sample5 | 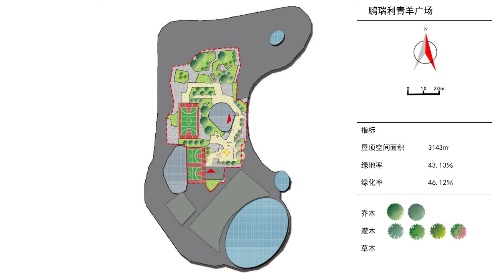  Recreation and Sightseeing | sample15 | 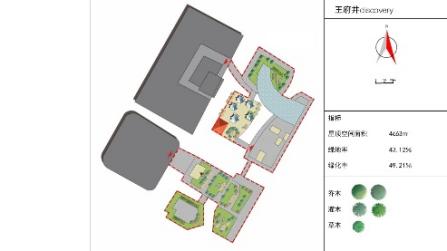  Catering and Entertainment | sample24 | 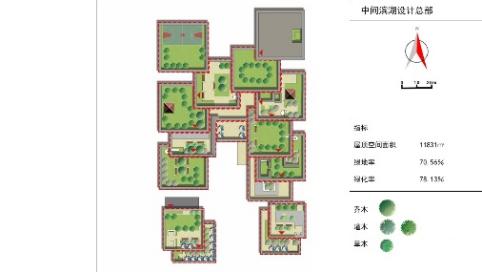  Recreation and Sightseeing |
| sample6 | 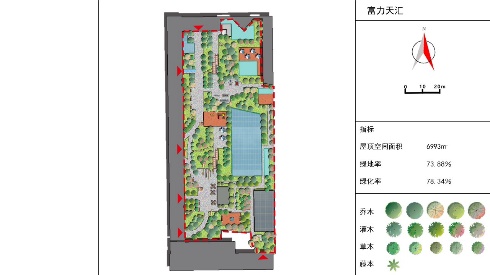  Recreation and Sightseeing | sample16 | 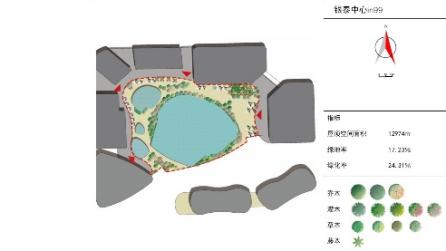  Catering and Entertainment | sample25 | 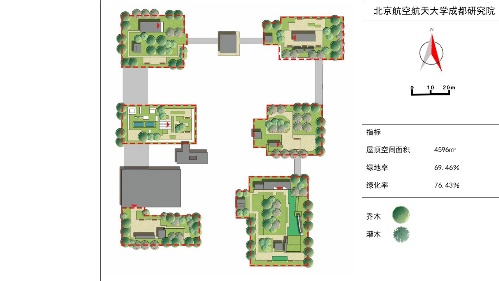  Recreation and Sightseeing |
| sample7 | 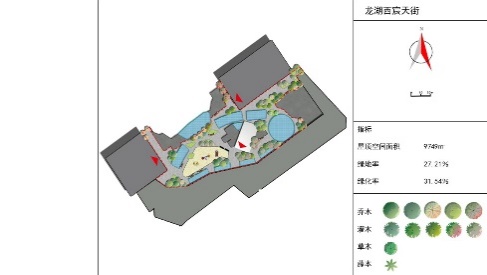  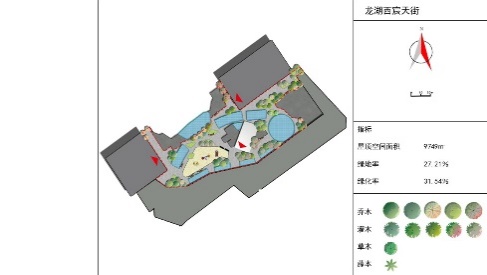  Recreation and Sightseeing | sample17 | 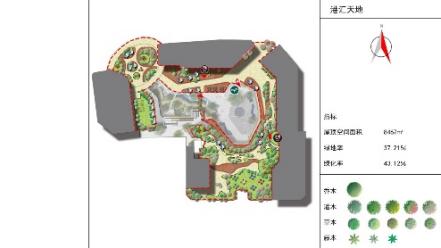  Sports and Fitness | sample26 | 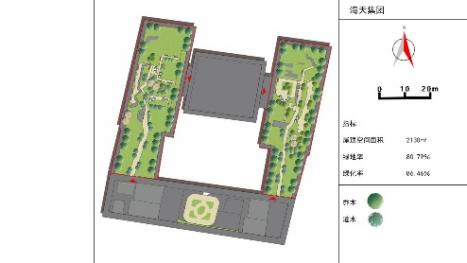  Recreation and Sightseeing |
| sample8 | 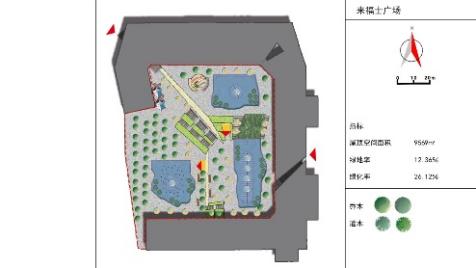  Sports and Fitness | sample18 | 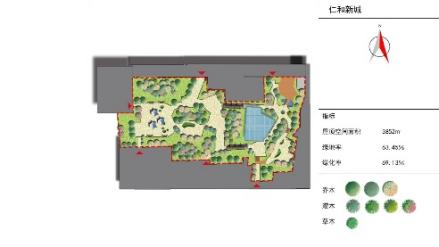  Catering and Entertainment | sample27 | 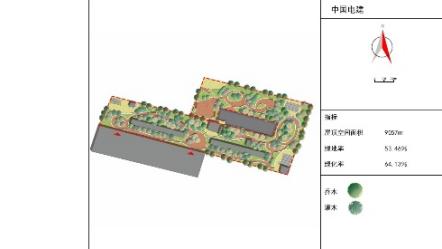  Recreation and Sightseeing |
| sample9 | 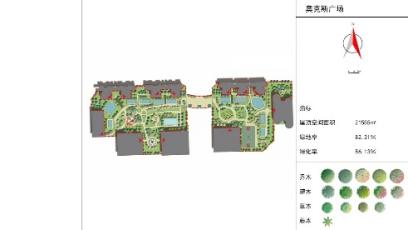  Recreation and Sightseeing | sample19 | 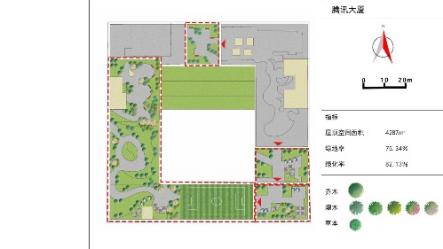  Recreation and Sightseeing | sample28 | 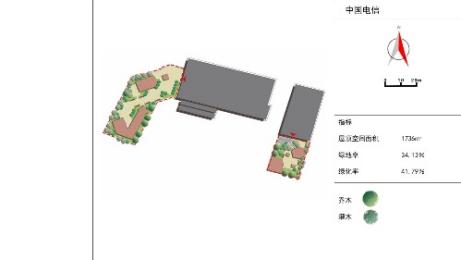  Sports and Fitness |
| sample10 | 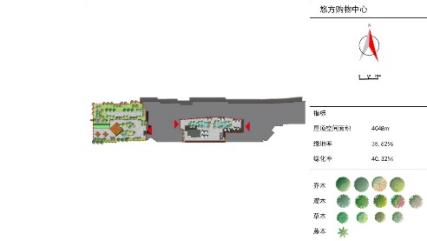Sports and Fitness |  |  |  |  |

Table S7 Numerical Values Corresponding to Experimental Model

|  | Functional Type |  | Green view Rate |  | Sky view factor |
| --- | --- | --- | --- | --- | --- |
| A1 | Recreation and Sightseeing | B1 | 32.79%， | C1 | 27.47% |
| A2 | Catering and Entertainment | B2 | 19.84% | C2 | 17.43% |
| A3 | Sports and Fitness | B3 | 10.54% | C3 | 9.19% |

Table S8 Expression of psychological indicators(EEG data)

| Indicators | Expression |
| --- | --- |
| Attention | Attention measures the degree of focused concentration on a specific task, reflecting both the depth of focus and the frequency of attention shifts. A higher attention score denotes stronger sustained concentration, whereas frequent attention shifts indicate distraction or cognitive overload. In the context of restorative environments, lower attention values are associated with mental relaxation, enabling individuals to disengage from work-related demands and relieve cognitive tension. |
| Engagement | Engagement represents a state of alertness and conscious absorption in task-related stimuli. It indicates the extent of cognitive and emotional immersion in a given activity. Higher engagement scores suggest greater involvement and workload intensity. Conversely, lower engagement values reflect a relaxed state conducive to psychological recovery, allowing mental detachment from task-oriented focus. |
| Excitement | Excitement denotes a positive form of physiological arousal characterized by activation of the sympathetic nervous system, including increased heart rate, muscle tension, and pupil dilation. Elevated excitement values correspond to stronger sympathetic activation and heightened alertness. From a restorative perspective, lower excitement values are favorable, indicating reduced physiological arousal and a transition toward calmness. |
| Stress | Stress measures the degree of tension or discomfort experienced in performing a task. Moderate stress can enhance alertness and short-term performance, but excessive stress impairs well-being and cognitive function. An optimal restorative rooftop environment is expected to lower stress levels, promoting comfort and psychological stability. |
| Relaxation | Relaxation reflects the capacity to disengage attention and recover from sustained concentration. High relaxation scores indicate successful restoration of cognitive resources and mental composure. Environments with abundant greenery and openness typically elevate relaxation levels, facilitating recovery from attentional fatigue. |
| Interest | Interest measures the degree of emotional attraction or aversion toward a stimulus, often described as affective valence. Lower interest scores represent indifference or aversion, while higher scores indicate positive emotional affinity. In restorative settings, elevated interest values signify that the environment elicits curiosity, enjoyment, or aesthetic appreciation, enhancing users’ emotional engagement with the space. |

Table S8 Expression of Social Health Indicators

| Indicators | Expression |
| --- | --- |
| Sense of Belonging | This indicator measures the degree of social connectedness an individual experiences within an environment. It reflects the extent to which people feel accepted, included, and integrated into a social group. Higher values indicate stronger perceived belonging, which contributes to emotional stability and psychological restoration. |
| Sense of Support | This indicator reflects an individual’s perception of the availability and reliability of assistance from others. It captures feelings of being valued and cared for, serving as a proxy for perceived social support. Environments that promote interaction and mutual aid typically enhance this sense, reinforcing social resilience. |
| Sense of Trust | This measures an individual’s confidence in others and in the surrounding social or physical environment. It reflects expectations of goodwill, reliability, and safety in interpersonal or communal relations. High trust levels are associated with reduced social anxiety and greater emotional comfort, key components of restorative experience. |
| Sense of Pleasure | This indicator evaluates the degree of positive affect and enjoyment individuals experience when engaging in social interaction or spending time in shared spaces. A high sense of pleasure reflects feelings of warmth, comfort, and happiness during social contact, reinforcing relaxation and satisfaction within restorative environments. |
| Sense of Safety | This assesses the extent to which individuals feel secure, free from fear, and confident in their surroundings. High safety perception minimizes environmental stress and supports a calm psychological state, thereby enhancing the overall restorative potential of the environment. |

Table S10 The Relationship Between Functional Types and Psychological Health Benefits

| ANOVA | | | | | | |
| --- | --- | --- | --- | --- | --- | --- |
|  | | sum of squares function | Degree of freedom | Mean square | F | Statistical significance |
| Attention | Intergroup differences | 4.810 | 2 | 2.405 | 1.771 | .192 |
|  | Intra-group differences | 32.600 | 24 | 1.358 |  |  |
|  | Total | 37.411 | 26 |  |  |  |
| Engagement | Intergroup differences | 1.281 | 2 | 0.641 | 0.674 | 0.519 |
|  | Intra-group differences | 22.832 | 24 | 0.951 |  |  |
|  | Total | 24.113 | 26 |  |  |  |
| **Excitement** | **Intergroup differences** | **34.269** | **2** | **17.135** | **6.516** | **0.005** |
|  | **Intra-group differences** | **63.106** | **24** | **2.629** |  |  |
|  | **Total** | **97.375** | **26** |  |  |  |
| Stress | Intergroup differences | 0.633 | 2 | 0.317 | 0.163 | 0.851 |
|  | Intra-group differences | 46.684 | 24 | 1.945 |  |  |
|  | Total | 47.318 | 26 |  |  |  |
| Relaxation | Intergroup differences | 6.794 | 2 | 3.397 | 2.409 | 0.111 |
|  | Intra-group differences | 33.842 | 24 | 1.410 |  |  |
|  | Total | 40.635 | 26 |  |  |  |
| **Interest** | **Intergroup differences** | **16.771** | **2** | **8.386** | **17.168** | **0.000** |
|  | **Intra-group differences** | **11.723** | **24** | **0.488** |  |  |
|  | **Total** | **28.494** | **26** |  |  |  |

Table S11 The Relationship Between Functional Types and Physical Health Benefits

| ANOVA | | | | | | |
| --- | --- | --- | --- | --- | --- | --- |
|  | | sum of squares function | Degree of freedom | Mean square | F | Statistical significance |
| Waling | Intergroup differences | .024 | 2 | 0.012 | .231 | 0.795 |
|  | Intra-group differences | 1.234 | 24 | 0.051 |  |  |
|  | Total | 1.258 | 26 |  |  |  |
| **Exerciese** | **Intergroup differences** | **8.542** | **2** | **4.271** | **218.467** | **0.000** |
|  | **Intra-group differences** | **.469** | **24** | **0.020** |  |  |
|  | **Total** | **9.011** | **26** |  |  |  |
| **Eating and Drinking** | **Intergroup differences** | **4.449** | **2** | **2.224** | **57.867** | **0.000** |
|  | **Intra-group differences** | **.923** | **24** | **0.038** |  |  |
|  | **Total** | **5.371** | **26** |  |  |  |
| **Viewing** | **Intergroup differences** | **3.294** | **2** | **1.647** | **18.981** | **0.000** |
|  | **Intra-group differences** | **2.082** | **24** | **0.087** |  |  |
|  | **Total** | **5.376** | **26** |  |  |  |
| **Conversation** | **Intergroup differences** | **1.241** | **2** | **0.620** | **7.686** | **0.003** |
|  | **Intra-group differences** | **1.937** | **24** | **0.081** |  |  |
|  | **Total** | **3.178** | **26** |  |  |  |
| **Reading** | **Intergroup differences** | **2.048** | **2** | **1.024** | **27.395** | **0.000** |
|  | **Intra-group differences** | **.897** | **24** | **0.037** |  |  |
|  | **Total** | **2.946** | **26** |  |  |  |

Table S12 The Relationship Between Functional Types and Social Health Benefits

| ANOVA | | | | | | |
| --- | --- | --- | --- | --- | --- | --- |
|  | | sum of squares function | Degree of freedom | Mean square | F | Statistical significance |
| Belonging | Intergroup differences | .025 | 2 | 0.013 | 0.188 | 0.830 |
|  | Intra-group differences | 1.615 | 24 | 0.067 |  |  |
|  | Total | 1.640 | 26 |  |  |  |
| Supporting | Intergroup differences | 0.008 | 2 | 0.004 | 0.076 | 0.927 |
|  | Intra-group differences | 1.298 | 24 | 0.054 |  |  |
|  | Total | 1.306 | 26 |  |  |  |
| Trust | Intergroup differences | 0.037 | 2 | 0.018 | 0.336 | 0.718 |
|  | Intra-group differences | 1.307 | 24 | 0.054 |  |  |
|  | Total | 1.344 | 26 |  |  |  |
| Pleasure | Intergroup differences | 0.017 | 2 | 0.008 | 0.155 | 0.857 |
|  | Intra-group differences | 1.313 | 24 | 0.055 |  |  |
|  | Total | 1.330 | 26 |  |  |  |
| Safety | Intergroup differences | 0.159 | 2 | 0.080 | 1.146 | 0.335 |
|  | Intra-group differences | 1.668 | 24 | 0.070 |  |  |
|  | Total | 1.828 | 26 |  |  |  |

Table S13 Data of the Combined Effects of Environmental Characteristics

| Variables | Psychological Health Benefits | Physical Health Benefits | Social Health Benefits |
| --- | --- | --- | --- |
| Catering and Entertainment | 0.012** | 0.007 | -0.028 |
|  | (2.48) | (0.60) | (-0.55) |
| Sports and Fitness | -0.004** | 0.088** | -0.047 |
|  | (-2.17) | (2.83) | (-0.80) |
| Medium GVI | 0.005* | 0.294** | 0.291*** |
|  | (1.99) | (3.16) | (5.43) |
| High GVI | -0.025 | 0.504*** | 0.572*** |
|  | (-1.68) | (7.68) | (9.89) |
| Medium SKF | 0.019** | 0.081** | 0.095*** |
|  | (2.03) | (2.59) | (3.21) |
| High SKF | 0.107** | 0.089** | 0.112*** |
|  | (2.18) | (2.49) | (3.49) |
| Catering and Entertainment*Medium GVI | 0.022 | 0.144 | 0.057 |
|  | (0.492) | (1.29) | (0.88) |
| Catering and Entertainment*  High GVI | 0.207** | 0.167* | 0.118* |
|  | (2.24) | (2.10) | (1.98) |
| Sports and Fitness*Medium GVI | -0.005 | 0.075 | 0.041 |
|  | (-0.18) | (0.74) | (0.58) |
| Sports and Fitness*High GVI | 0.033 | 0.069* | 0.042** |
|  | (1.01) | (1.93) | (2.06) |
| Catering and Entertainment*Medium SKF | 0.002** | -0.035 | 0.063 |
|  | (2.07) | (-0.40) | (1.17) |
| Catering and Entertainment*High SKF | -0.019 | 0.163** | 0.126* |
|  | (-0.68) | (2.33) | (1.98) |
| Sports and Fitness*  Medium SKF | 0.007 | -0.005 | -0.011 |
|  | (0.49) | (-0.07) | (-0.16) |
| Sports and Fitness*High SKF | 0.043** | 0.149** | 0.078 |
|  | (2.49) | (2.65) | (0.16) |
| Medium GVI*Medium SKF | -0.032 | -0.085 | -0.083 |
|  | (-1.55) | (-0.93) | (-1.31) |
| Medium GVI*High SKF | 0.048** | 0.043 | 0.014 |
|  | (2.60) | (0.46) | (0.24) |
| High GVI*Medium SKF | 0.078 | -0.035 | -0.055 |
|  | (1.31) | (-0.37) | (-0.81) |
| High GVI*High SKF | 0.004** | 0.039** | 0.074** |
|  | (2.41) | (2.55) | (2.42) |
| _cons | 0.998*** | 3.343*** | 3.491*** |
|  | (3.61) | (4.74) | (6.05) |
| N | 147 | 147 | 147 |
| R^2^ | 0.468 | 0.503 | 0.463 |

(Note: The values in parentheses are t-values; * indicates p < 0.1, ** indicates p < 0.05, *** indicates p < 0.001)

| 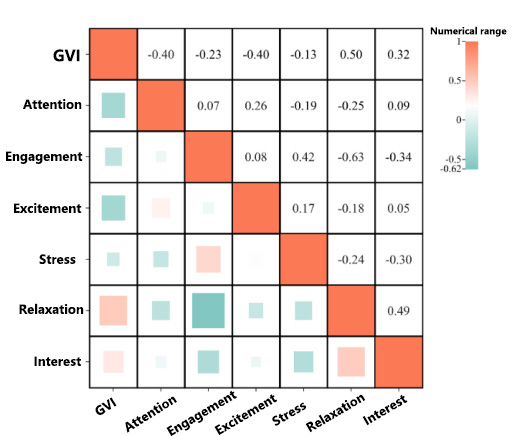  **Figures S1** The Correlation Between Green View Rate and psychological health Benefits | 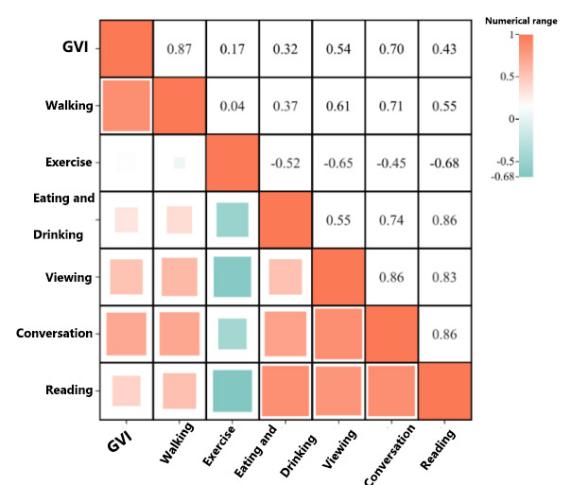  **Figures S2** The Correlation Between Green View Rate and physical health Benefits |
| --- | --- |

| 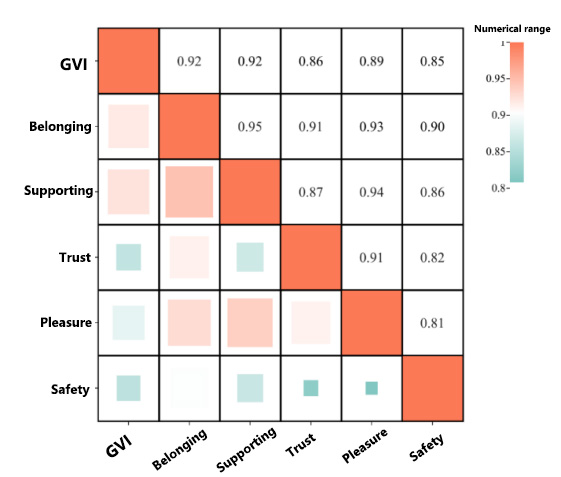  **Figures S3** The Correlation Between Green View Rate and social health Benefits | 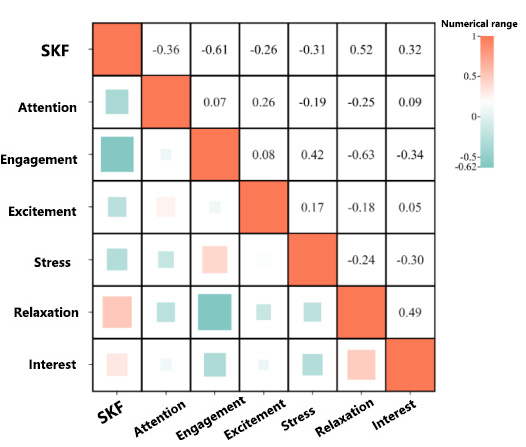  **Figures S4** The Correlation Between SKY View Factor and psychological Health Benefits |
| --- | --- |

| 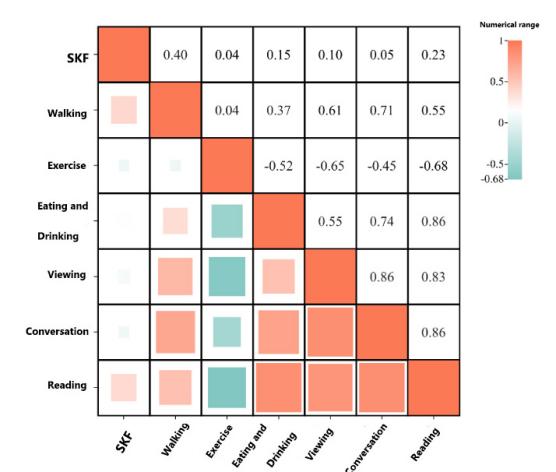  **Figures S5** The Correlation Between SKY View Factor and physical health Benefits | 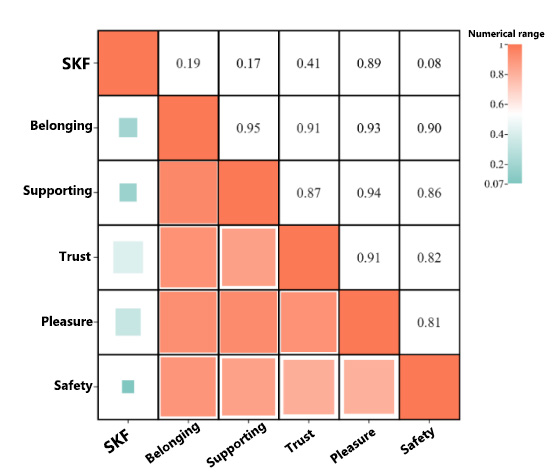  **Figures S6** The Correlation Between SKY View Factor and social health Benefits |
| --- | --- |
